# A novel therapeutic approach targeting PD-L1 in HNSCC and bone marrow-derived mesenchymal stem cells hampers pro-metastatic features in vitro: perspectives for blocking tumor-stroma communication and signaling

**DOI:** 10.1186/s12964-025-02073-7

**Published:** 2025-02-10

**Authors:** Ylenia Ferrara, Debora Latino, Angela Costagliola di Polidoro, Angela Oliver, Annachiara Sarnella, Maria Grazia Caprio, Laura Cerchia, Menotti Ruvo, Annamaria Sandomenico, Antonella Zannetti

**Affiliations:** 1 Istituto di Biostrutture e Bioimmagini-CNR, Napoli, Italy; 2 Telethon Institute of Genetics and Medicine, Pozzuoli, Italy; 3 Università della Campania “L. Vanvitelli”, Caserta, Italy; 4 Istituto degli Endotipi in Oncologia, Metabolismo e Immunologia “G. Salvatore”, CNR, Napoli, Italy

## Abstract

**Background:**

Current conventional treatment regimens for head and neck squamous cell carcinoma (HNSCC), are poorly effective because of the emergence of resistance mechanisms. Many studies have reported how the tumor microenvironment influences tumor response to immune checkpoint inhibitors targeting PD-1/PD-L1. It has been reported that overexpression of PD-L1 correlates with and is involved in cancer progression by promoting epithelial-to-mesenchymal-transition (EMT) program, stemness and tumor cell invasiveness through AKT and MAPK pathways. In this study, we investigated how bone marrow mesenchymal stem cells (BM-MSCs) recruited and educated by HNSCC cells are able to promote tumor cell invasion and EMT program. In addition, we analyzed how the crosstalk between stromal cells and tumor cells can affect PD-L1 expression levels. In this context, we developed and characterized a novel anti-PD-L1 recombinant Fab (rFab’) and tested its ability to potentiate the effect of cisplatin.

**Methods:**

BM-MSCs and HNSCC cells co-cultures, cell migration and invasion were performed using Boyden chambers. The effect of treatments on cell viability and growth were analyzed by MTT and clonogenic assay, respectively. The anti-PD-L1 rFab’ was prepared in *E. Coli* and tested for its binding on HNSCC cells and BM-MSCs by FACS analysis and fluorescence microscopy. PD-L1, p-AKT, p-ERK, N-cadherin and β-catenin expression levels were analyzed by western blotting.

**Results:**

BM-MSCs were induced by tumor cells to migrate, invade and to trans-differentiate in cancer associated fibroblasts (CAFs) as demonstrated by increased expression levels of α-SMA and FAP-α. BM-MSCs contributed to HNSCC invasiveness by increasing p-AKT, p-ERK, N-cadherin and β-catenin expression levels. When BM-MSCs and HNSCC cells were co-cultured the level of PD-L1 expression was enhanced in both cells indicating a reciprocal support in favoring tumor aggressiveness. Tumor cell treatment with rFab’ anti-PD-L1 reduced their viability, growth, migration and invasion and blunted the underlying signaling pathways. In addition, rFab’ anti-PD-L1 was able to potentiate the antitumor effect of cisplatin on HNSCC cells.

**Conclusions:**

BM-MSCs recruited and educated by HNSCC cells support tumor cell aggressiveness via PD-L1. A novel rFab’ anti-PD-L1 reduces HNSCC proliferation, migration and invasion and potentiates the cisplatin effect suggesting its potential to be conjugated with drugs for immuno-cytotoxic therapy.

**Supplementary Information:**

The online version contains supplementary material available at 10.1186/s12964-025-02073-7.

## Background

Head and neck squamous cell carcinomas (HNSCCs) represent up to 90% of the head and neck cancer, and are the sixth most common cancer worldwide [1]. Currently, the efficacy of conventional treatment regimens for HNSCC, including surgery and radiotherapy with/without conventional chemotherapy, is not satisfactory due to the emergence of drug resistance mechanisms [1]. Therefore, many studies are focusing on the use of immunotherapy with immune checkpoint inhibitors (ICIs), such as those targeting PD-1/PD-L1 in HNSCC treatment. High expression levels of PD-L1 have been found in many HNSCCs suggesting the crucial role of this pathway on tumor immune evasion [2]. In addition, recent studies have revealed a temporal heterogeneity of PD-L1 status during HNSCC progression [3]. Results from clinical trials using ICIs in this cancer have shown that only a subgroup of patients really benefits from treatment [4], underlying the importance of understanding the mechanisms involved in immuno resistance to improve immuno response. Interestingly, the overexpression of PD-L1 does correlate and is involved in supporting cancer progression by promoting epithelial-to-mesenchymal-transition (EMT) program, together with stemness and tumor cell invasiveness through PI3K-AKT and MAPK pathways [5]. Through activation of these mechanisms tumor cells undergo a global change in cell architecture, leading to the loss of cell-cell adhesions in favor of cell-extracellular matrix (ECM) interactions and cell migration/invasion thus acquiring a pro-metastatic phenotype with an increased potential to spread from the primary tumor mass [6]. Recently, the immune-independent PD-L1 functions have gained increasing attention. It has been reported that PD-L1 promotes HNSCC proliferation in vitro and in vivo through mTOR signaling [7]. Despite numerous studies have reported how the tumor microenvironment (TME) influences the tumor response to ICIs, a deeper understanding of its role in regulating immune responses in HNSCC would be needed, as it could better predict the behavior of this tumor toward PD-L1/PD-1-targeted therapies [8]. In TME stromal cells and tumor cells communicate each other through multiple signals [9]. Naïve mesenchymal stem cells (MSCs) are recruited by tumor cells, including HNSCC cells, through the release of various chemokines, cytokines and growth factors and are ‘educated’ to support tumor aggressiveness, favoring, in various cancer types, the emergence of mechanisms involved in drug resistance, metastasis and immune evasion, such as EMT and CSCs [9–11]. Education of stromal cells is a dynamic process where non-malignant cells progressively switch from a neutral or antitumorigenic role toward a pro-tumorigenic role [11]. Interestingly, it has been shown that myofibroblasts and MSCs derived from the bone marrow directly favor the metastatic spread of cancer cells [9]. It has been also observed that MSCs are increased in HNSCC tumors compared to normal tissue [12], show a phenotype similar to normal BM-MSCs and are able to inhibit T cell proliferation. Here, we have investigated how BM-MSCs can be recruited and transformed into cancer associated fibroblasts (CAF)-like by HNSCC cells and how cross-talk between tumor and stromal cells supports the onset of a pro-metastatic state by increasing tumor cell migration, invasiveness and stemness as well as EMT program by involving PD-L1 through AKT and MAPK signaling pathways. Recently, several studies have revealed the importance of combining immunotherapy targeting the PD-L1 signal with conventional, targeted therapies to improve treatment outcomes through a synergistic effect, exercising their complementary advantages [13–15]. Therefore, we have generated and characterized a novel rFab’ anti-PD-L1 and demonstrated its ability to suppress proliferation, migration and invasion of HNSCC cells by inhibiting p-AKT and p-ERK as well as EMT and to enhance the cytotoxic effect of cisplatin.

## Methods

### Cell lines and culture conditions

The head and neck squamous carcinoma cell lines, FaDu and SCC-011, were purchased from the American Type Culture Collection (ATCC, Manassas, VA) and Cellosaurus JHU-011 (CVCL_5986), respectively. FaDu cells were cultured in Dulbecco Minimum Essential Medium (DMEM) whereas SCC-011 cells in the Roswell Park Memorial Institute (RPMI), supplemented with 10% fetal bovine serum (FBS) and 1% L-glutamine-penicillin–streptomycin and grown at 37 °C with 5% CO_2_. BM-MSCs were purchased from Lonza (PT-2501) and were grown in MSCGM™ Mesenchymal Stem Cell Growth Medium (PT-3001) in a humidified incubator in 5% CO_2_ at 37 °C. BM-MSCs passed the quality inspection conducted by Lonza company using cell viability (more than 75%), adipogenic and osteogenic differentiation (Oil Red O staining and calcium deposition staining) and FACS analysis of cell surface markers (more than 90% were positive for CD29, CD44, CD105 and CD166, and negative for CD14, CD34 and CD45).

### Co-culture assay

FaDu and SCC-011 cells were co-cultured with BM-MSCs and vice versa using Boyden chambers with microporous-3 μm membrane (Corning, NY). To this aim, BM-MSCs (1 × 10^5^), FaDu and SCC-011 cells (2 × 10^5^) were seeded into a 6-multiwell. After 24 h, FaDu and SCC-011 cells and BM-MSCs (2 × 10^5^) were added to the top of respective chambers for 72 h.

### Cell viability assay

The viability of FaDu, SCC-011 and BM-MSCs (4 × 10^3^ cells/well, 96-well plates), treated with rFab’ anti-PD-L1 (200 nM) alone or in combination with 1 µM cisplatin (Cis-Pt), was assessed with CellTiter 96 AQueous One Solution Cell Proliferation Assay (Promega BioSciences Inc., Fitchburg, WI, USA) using 3-(4,5-dimethylthiazol-2yl)-5-(3-carboxymethoxy-phenyl)-2-(4-sulfophenyl)-2 H tetrazolium (MTS), according to the manufacturer’s instructions and as previously described [16].

### Cell migration assay

Cell migration was performed using 24-well Boyden chambers (Corning, NY) with microporous-8 µm membrane [17]. BM-MSCs (0.5 × 10^5^ cells/well in 100 µL of serum-free medium) were treated with or without Cis-Pt (1 µM) and rFab’ anti-PD-L1 (200 nM) and seeded in the upper chamber in the presence of different chemo-attractants in the lower chamber (10% FBS and 1.5 × 10^5^ FaDu/SCC-011). After incubation for 24 h in a humidified incubator in 5% CO2 at 37 °C, the non-migrated cells were removed with cotton swabs, whereas the cells that had migrated were visualized by staining the membrane with 0.1% crystal violet in 25% methanol. 10 random fields/filter were counted under a phase contrast microscope (Leica) and images were captured using a digital camera (Canon). All experiments were performed at least three times. All the results are expressed as the percentage of migrating cells considering the vehicle control sample as 100%.

### Cell invasion assay

The cell invasion assay was performed using the Boyden chamber with microporous-8 µm membrane coated with 50 µL of diluted Matrigel (1:5 in PBS) (Corning, NY, USA). FaDu, SCC-011 cells and BM-MSCs (1 × 10^5^ and 0.5 × 10^5^ cells/well in 100 µL serum-free medium) were treated with or without rFab’ anti-PD-L1 (200 nM) alone or in combination with Cis-Pt (1 µM), and placed in the top chamber in the presence of different chemo-attractants in the lower chamber (10% FBS and1.5 × 10^5^ FaDu/SCC-011, 0.5 × 10^5^ BM-MSCs). Cells were allowed to migrate to invade Matrigel for 72 h in a humidified incubator with 5% CO_2_ at 37 °C. To visualize and analyze invading cells, the same experimental procedure described above for cell migration assay was performed. All experiments were performed at least three times.

### Spheroid formation of HNSCC cells

FaDu and SCC-011 cells (1 × 10^4^cell/well) were seeded in ultra-low attachment 96-multiwell-plates (Corning) and grown in spheroid medium containing serum-free DMEM supplemented with B27 (1X), bFGF (20 ng/mL) and EGF (10 ng/mL). Cells were incubated at 37°C with 5% CO_2_ for 48 hours for allowing spheroid formation. Then, spheroids were treated with or without rFab’ anti-PD-L1 (200 nM) alone or in combination with Cis-Pt (1 µM) for 72 h. Spheroid formation was analyzed under a phase-contrast microscopy and the size and number of formed spheroids were calculated using ImageJ.

### 3D cell viability assay

The viability of 3D spheroids from FaDu and SCC-011 treated with anti-PD-L1 rFab’ (200 nM) alone or in combination with Cis-Pt (1 µM), was assessed with CellTiter-Glo^®^ 3D (Promega BioSciences Inc., Fitchburg, WI, USA) according to the manufacturer’s instructions.

### Immunofluorescence staining of 3D spheroids

FaDu and SCC-011 cells were seeded in ultra-low attachment 6-multiwell-plates (Corning) and grown as described above to obtain 3D spheroids. After washing with PBS, 3D spheroids were fixed in 4% paraformaldehyde in PBS for 20 min at room temperature, then were blocked for 1 hour in 5% bovine serum albumin (BSA) in PBS. Spheroids were labelled with rFab’ anti-PD-L1 (200 nM) and FITC-Atezolizumab (200 nM) in 5% BSA and incubated overnight at 4 °C and 1 h at RT, respectively. Spheroids with rFab’ anti-PD-L1 were labelled with anti-human IgG (Fab specific) − FITC antibody (Sigma - F5512) at a dilution of 1:50 for 1 h at RT in the dark. Nuclei were stained by DAPI (4′,6-diamidin-2-phenylindol) (Sigma-Aldrich, Taufirchen, Germany) for 30 min and washed with DPBS. For evaluation, 3D spheroids were analysed with Nikon eclipse Ti2 microscopy using the NIS-elements AR software (Nikon Instruments Inc., Melville, NY, USA).

### Clonogenic Assay

FaDu and SCC-011 cells (500 cells/well, 6-well plates) were cultured with rFab’ anti-PD-L1 (200 nM) alone or in combination with Cis-Pt (1 µM) at 37 °C for 10 days. After washes with DPBS, cells were fixed and stained with 0.1% crystal violet in 25% methanol. Following 30 min at RT, culture dishes were washed with DPBS and colonies were photographed. 1% Sodium Dodecyl Sulphate was added on the cells perfectly washed, in order to induce crystal violet dissolution. Absorbance was recorded at 490 nm by a 96-well-plate ELISA reader.

### Western blot analysis

Cells were lysed in lysis buffer (40 mM HEPES pH 7.5, 120 mM NaCl, 5mM MgCl2, 1mM EGTA, 0.5 mM EDTA, e 1% Triton X-100) supplemented with phosphatases (20 mM α-glycerol-3-phosphate e 2.5 mM Na-pyrophosphate) and proteases (Complete Tablets, EDTA-free, Roche) inhibitors. After lysis, cells were centrifuged 20 min at 13,200 RPM and 4 °C. Proteins were quantified by Bradford assay (Biorad, protein assay). An equal amount of proteins from cells was separated by 4–12% SDS-PAGE and was transferred to a nitrocellulose membrane. Blots were blocked for 1 h with 5% non-fat dry milk and then incubated over night with the following primary antibodies: anti-N-cadherin (CST 13116, Cell Signaling Technology Inc), anti-β-catenin (CST-9782;), anti-AKT (CST-9272), anti-pAKT (CST-9271), anti-ERK (CST-9102), anti-pERK (CST-9101), anti-PD-L1 (CST-13684), anti-α-SMA (ab5694, Abcam), anti-FAP-α (sc-100528, Santa Cruz Biotechnology), anti-GAPDH (G8795, Sigma), anti-Tubulin (sc-5286,) and anti-Actin (A4700, Sigma). After washing with 0.1% Tween-20 in PBS, filters were incubated with their respective secondary antibodies for 1 h and analyzed using the enhanced chemiluminescence (ECL) system. Densitometric analyses were performed on at least two different expositions to assure the linearity of each acquisition using ImageJ software (v1.46r).

### In silico analysis of the expression of PD-L1

For PD-L1 mRNA expression analysis in HNSCC patients the Genomics Analysis and Visualization platform (R2: Genomics analysis and visualization platform; http://r2.amc. nl) was used. The analysis was performed with the following datasets: GSE18674 that includes 22 human normal tissues and GSE42743 that includes 103 oral cavity tumors from Stanford University School of Medicine, Stanford Cancer Center (Standford, CA, USA). The correlation was assessed by one-way analysis of variance (ANOVA), through the R2 platform and presented in box plots.

### Preparation and characterization of anti-PD-L1 recombinant Fab’ (rFab’)

The Fab’ fragment of Atezolizumab (ATE, https://go.drugbank.com/drugs/DB11595*)* was prepared in *E. Coli* as recombinant protein using a plasmid *ad hoc* engineered. The C-terminus of the Fab’ heavy chain included the CPP residues of the hinge region together with the peptide GSGALQPTQGAMPA that is a microbial Transglutaminase (MTG)-sensitive consensus site [18–20]. The plasmid was purchased from GenScript (USA). The rFab’ expression was optimized in BL21 (DE-3) *E. Coli* strain (Invitrogen, Waltham, Massachusetts, United States) using 2xYT medium and inducing with 2 mM IPTG, at 16 °C for 16 h. The bacterial cultures were harvested by centrifugation and the resulting pellet was washed with PBS buffer and stored at -80 °C. The periplasmatic rFab’ extraction was performed adding lysis buffer containing 100 mM Tris-HCl pH 7.4, 10 mM EDTA pH 8.0 and the protease inhibitor cocktail (Complete EDTA free tablet, Roche, Basel, Switzerland). After overnight incubation at 30 °C in an orbital shaker under constant agitation at 250 rpm, the mixture was centrifugated at 12,000 rpm for 30 min at 4 °C and the lysate was again clarified by filtration on 0.2 μm sterile filters before injection onto the CaptureSelect™ IgG-CH1 affinity matrix column (Thermo Fisher Scientific, Milano, Italy). The purification was carried out using an AKTA Purifier system (GE Healthcare, Milan, Italy) at a flow rate of 0.8 mL/min monitoring the absorbance at 280 nm. The bound fractions containing the recombinant Fab were collected and immediately neutralised with 2 M Tris-HCl pH 9 (50 µL/mL) to maintain the optimal isotonic condition. The eluted fractions were pooled, buffer exchanged with PBS and concentrated using Amicon centricons with 30 kDa cut-off (Millipore, Burlington, Massachusetts, United States). The oligomeric state of rFab’ fragment was analysed by gel filtration on a Sephadex 200 column (GE Healthcare/Cytiva, Milan, Italy) in PBS buffer. The concentration was measured through determination of the absorbance at 280 nm (ε_280nm_ = 81415 M^− 1^cm^− 1^) using a NanoDrop 2000 C spectrophotometer. The purity was estimated by SDS-PAGE analysis.

### Structural characterization of rFab’ anti-PD-L1 by circular dichroism

Structural analyses by Circular Dichroism (CD) was conducted using a Jasco spectropolarimeter, J-710 model, equipped with a Peltier system to control temperature using 110-QS quartz cuvettes of 1.0 mm path length (Hellma, Mullheim, Baden, Germany). Samples were prepared at 0.2 mg/mL (4 µM) in 10 mM Sodium Phosphate, pH 7.5. Spectra were collected in the wavelength range 260–200 nm as reported elsewhere [18–20]. Thermal denaturation experiments were performed by monitoring changes in ellipticity at 218 nm by exposure to increasing temperatures between 20 °C and 100 °C, heating the samples at 60 °C/h (1 °C/min). The melting temperature was determined by the method of the first derivative. Final spectra were corrected by subtracting the corresponding baseline spectrum obtained under identical conditions and smoothed using the SpectraManager software, version 1.53 (Easton, MD, USA). Data were exported and charted using GraphPad vers 5 software (GraphPad Software, San Diego California USA).

### Surface Plasmon Resonance binding assays (SPR)

The recombinant Fab’ was tested for its ability to bind the target antigen *rh*PD-L1 protein (10084-H08H, Sinobiological) performing real-time binding assays by Surface Plasmon Resonance using CM5 sensor chips and a Biacore 3000 instrument (GE Healthcare, Milan, Italy) as previously reported [21–24]. *rh*PD-L1 was immobilized on a CM5 sensor chip (GE Healthcare, Milan, Italy) using the standard amine coupling chemistry at 10 µg/mL in 10 mM NaAc, at pH 4.5 achieving three different levels of immobilization (low, medium and high). A reference channel used as blank was prepared performing the same procedure without ligand. Atezolizumab used as reference and the corresponding rFab’ anti PD-L1 fragment were tested at increasing concentrations between 0.075 nM and 4.0 nM using HBS-EP (10 mM HEPES, pH 7.4, 150 mM NaCl, 3 mM EDTA, 0.005% surfactant P20) as running buffer. All analyses were carried out at 25 °C, at the constant flow rate of 20 µL/min, injecting 60 µL (3 min contact time) of analyte solutions. NaOH at concentrations ranging between 5 mM and 20 mM was used to regenerate the chip surface. For every single analysis, experimental sensorgrams were aligned, subtracted of blank signals and overlaid. All mathematical manipulations and fitting were performed using the BiaEvaluation software, version 4.1 from GE Healthcare. The data provided a good fit when processed assuming a 1:1 Langmuir binding model. Sensorgrams were exported and charted using GraphPad vers. 5 Software (GraphPad Software, San Diego California USA).

### Site specific rFab’ labelling via MTG

As proof of concept, the rFab’ anti-PD-L1 bearing at the C-terminus of the heavy chain the fragment GSGALQPTQGAMPA, was site specifically labelled with the linker Ac-Biotin-βA-KAYA-CONH_2_ (MW_exp_ 731.37 Da/MW_theor_ 731.60 Da using microbial transglutaminase (MTG) as reported previously [18–20, 23].

Site-specific labelling reaction was optimized with the linker at molar ratio of 1:10 and MTG at 0.25 U/mL in 50 mM NaH_2_PO_4_ pH 7.4 at room temperature. The reactions were monitored over time using LC-ESI-TOF mass spectrometry.

### Peptide and protein identification by LC- ESI-TOF-MS analysis

Mass spectrometry analyses were performed with an Agilent 1290 Infinity LC System coupled to an Agilent 6230 TOF. The liquid chromatographic Agilent 1290 LC module was coupled with a PDA detector and a 6230 time-of-flight MS detector, along with a binary solvent pump degasser, column heater and autosampler. Solvents were: A, 0.05% TFA/H_2_O (v/v) and B, 0.05% TFA/CH_3_CN (v/v). Chromatographic analyses of peptides were performed using a C18 Biobasic column applying a linear gradient from 5 to 70% of solvent B in 10–20 min. Chromatographic analyses of recombinant Fab fragments under non-reducing and reducing conditions were performed using a C4 Biobasic column applying a linear gradient from 25 to 65% of solvent B for 20–30 min, flow rate 0.2 mL/minutes, with the heater at a constant temperature of 20 °C. UV spectra were monitored in the range between 200 nm and 600 nm, respectively. The mass analyzer Agilent 6230 TOF-MS was set to operate in positive ion scan mode with mass scanning from 100 to 3200 m/z. Nitrogen was used as the drying and nebulizer gas. The instrument acquired data using the following parameters: drying gas temperature, 325 °C; drying gas flow, 10 L/min; nebulizer, 20 psi; sheath gas temperature, 400 °C; sheath gas flow, 11 L/min; VCap. 3.500 V; nozzle, 0 V; fragmentor, 200 V; skimmer, 65 V; and octapole RF Vpp was 750 V. The instrument was set to extended dynamic range mode (2 GHz). Tuning and calibration were performed before sample runs. Data collection and integration were performed using MassHunter workstation software (version B.05.00). Data were stored in both centroid and profile formats during acquisition. A constant flow of Agilent TOF reference solution through the reference nebulizer allowed the system to continuously correct for any mass drift by using two independent reference lock-mass ions, purine (m/z 119.03632) and HP-922 (m/z 922.000725), to ensure mass accuracy and reproducibility. Target compounds were detected and reported from accurate mass scan data using Agilent MassHunter Qualitative software.

### Wound healing assay

FaDu and SCC-011 cells were cultured in 6-well plates at 4 × 10^5^ cells/well to achieve confluent monolayers. Straight wounds were made by using 200-µL pipette tips. After washing with medium to remove cell debris, wounded monolayers were treated with Cis-Pt (1 µM) and anti-PD-L1 rFab’ (200 nM) alone or in combination. The wound gaps were photographed at regular intervals (0, 24, and 48 h). The distance between the edges of the scratch was measured by ImageJ, the average distance was quantified and the extent of wound closure was determined as follows: Wound closure (%) = 1 − (wound width tx/wound width t0) × 100.

### Cell binding assay by flow cytometry analysis

FaDu and SCC-011 grown in 2D (3.5 × 10^5^ cells/sample) and 3D (spheroids) were incubated 15 minutes at room temperature with rFab’ anti-PD-L1 (200 nM) or FITC-ATE (200 nM), washed with Wash Buffer (2% FBS-PBS) and then incubated with anti-human IgG (Fab specific) − FITC antibody (Sigma - F5512) for 30 min on ice in the dark. The cells were washed and resuspended in 500 µL of wash buffer and then the samples were acquired using BD Accuri™ C6 Plus Flow Cytometer. Data analysis was performed using FlowJo 10 software (BD Biosciences). All final data represent specific binding, determined by subtraction of non-specific binding observed in assays performed with cells alone or incubated only with secondary FITC antibody.

## Statistical analysis

Data were analyzed with GraphPad Prism statistical software 8.0 (GraphPad Software, La Jolla, CA, USA). The results were obtained from at least three independent experiments and are expressed as means ± standard deviation, and significance was determined using Student’s t-test. A p-value < 0.05 was considered statistically significant.

## Results

### HNSCC cells induce recruitment and trans-differentiation of BM-MSCs

**Fig. 1:**
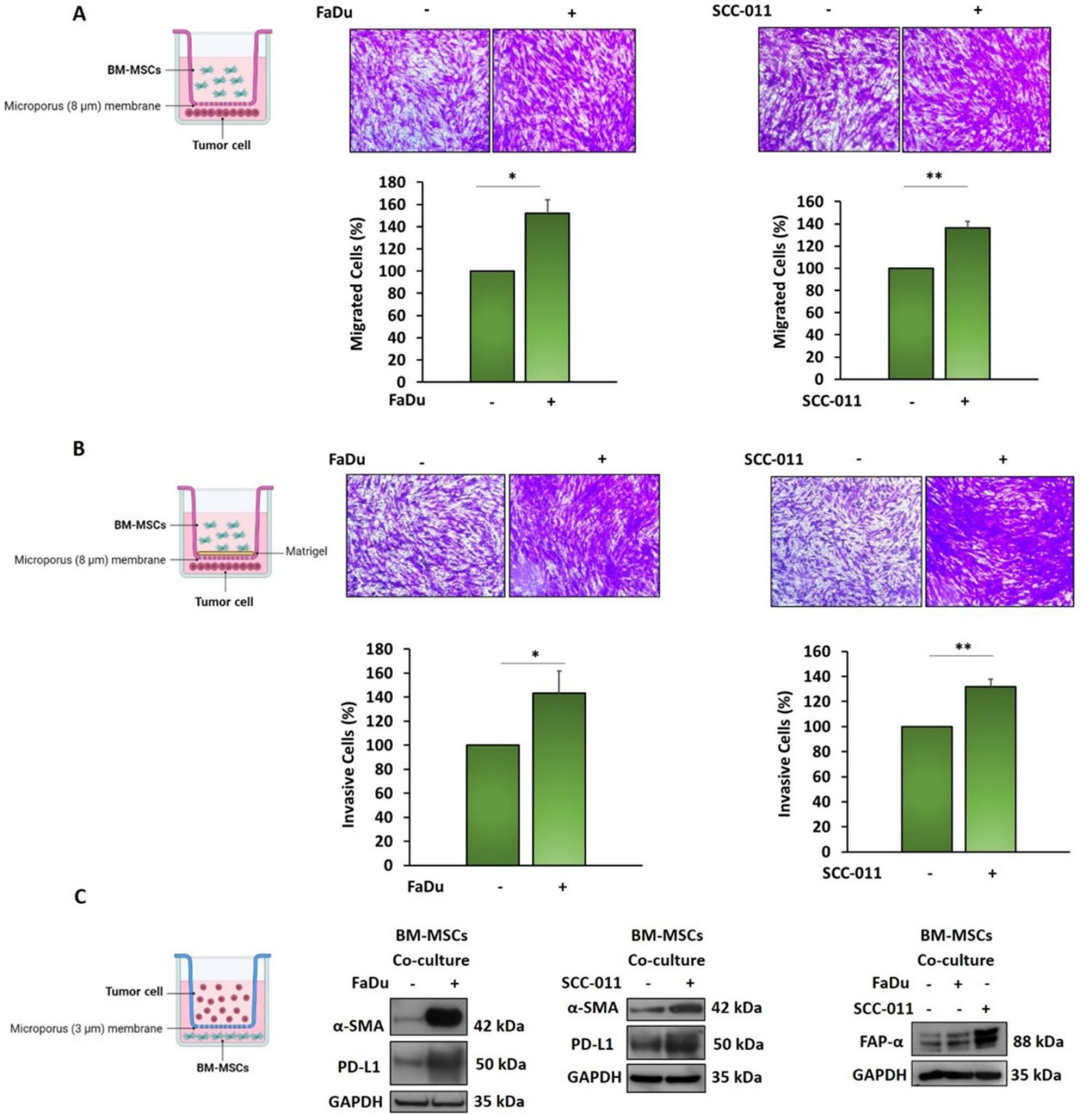
(**A**) Migration assay of BM-MSCs was performed using Boyden chamber containing inserts of polycarbonate membranes with 8 μm pores for 24 h. BM-MSCs were seeded in the upper chamber while FaDu and SCC-011 cells were seeded in the lower chamber, medium supplemented with FBS 10% was used as control. (**B**) Invasion assay of BM-MSCs was performed was performed using Boyden chamber containing inserts of polycarbonate membranes with 8 μm pores coated with Matrigel for 72 h as above described. All the data are expressed as percentages of migrating and invading cells considering the untreated control as 100%. Bars depict mean ± SD of three independent experiments (*** *p* < 0.0001; ** *p* < 0.001; * *p* < 0.01). (**C**) BM-MSCs were co-cultured for 72 h with FaDu and SCC-011 using a Boyden chamber containing inserts of polycarbonate membranes with 3 μm pores. The expression levels of PD-L1, α-SMA and FAP-α were analyzed by western blotting. GAPDH was used as loading control. Representative images are shown

Mesenchymal stem cells are recruited by tumor cells in TME for supporting their aggressiveness [9, 25]. Here, we evaluated the ability of BM-MSCs cells to migrate towards HNSCC cells (FaDu and SCC-011 cell lines) and invade the ECM (matrigel) using trans-wells, where BM-MSCs were seeded in the upper chamber while tumor cells were seeded in the lower chamber as chemoattractant. BM-MSCs showed a higher capacity to migrate (FaDu 52%, *p* < 0.01; SCC-011 37%, *p* < 0.001) and invade ECM (FaDu 43%, *p* < 0.01; with SCC-011 32%, *p* < 0.001) in presence of HNSCC cells compared to control (medium supplemented with FBS 10%) (Fig. 1A, B). In order to investigate the behavior of BM-MSCs and their impact in HNSCC TME we analyzed the cross-talk between these cells and HNSCC cells through co-culture experiments. When BM-MSCs were grown in presence of FaDu and SCC-011 cells, they showed increased expression levels of alpha smooth muscle actin (α-SMA) and fibroblast activation protein alpha (FAP-α) indicating their transformation in tumor-educated BM-MSCs [25] with features of CAFs (Fig. 1C).

### Cross-talk between BM-MSCs and HNSCC cells enhances tumor cell invasiveness, EMT program and PD-L1 expression levels

**Fig. 2:**
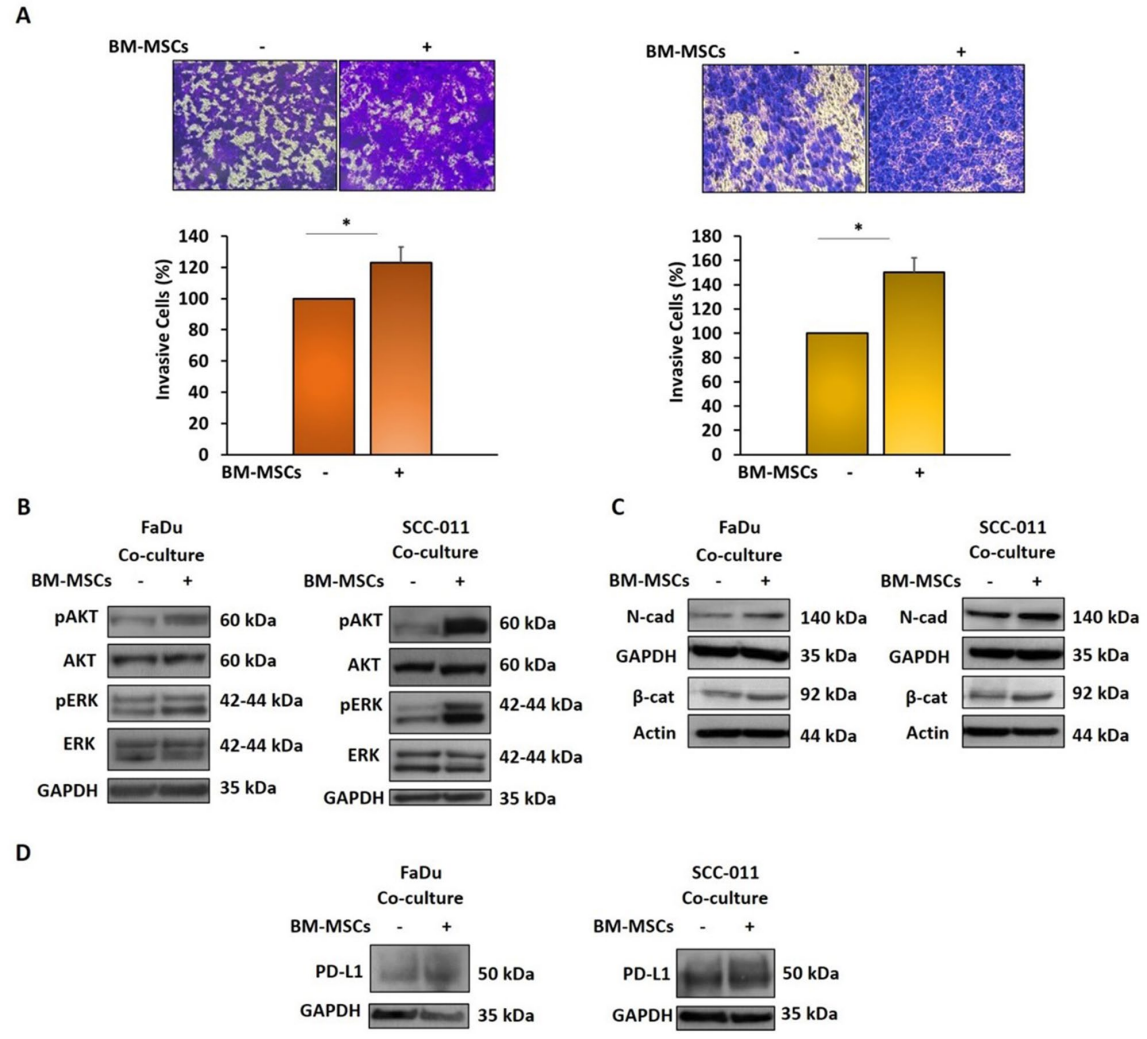
(**A**) Invasion assay of FaDu and SCC-011 was performed using Boyden chamber containing inserts of polycarbonate membranes with 8 μm pores coated with Matrigel for 72 h. Tumor cells were seeded in the upper chamber while BM-MSCs were seeded in the lower chamber, medium supplemented with FBS 10% was used as control. Representative images of at least three different experiments are shown. The results are expressed as percentage of invading cells considering the untreated control as 100% (** *p* < 0.001; * *p* < 0.01). FaDu and SCC-011 cells were co-cultured with BM-MSCs for 72 h using a Boyden chamber containing inserts of polycarbonate membranes with 3 μm pores and the expression levels of (**B**) pAKT/AKT and pERK/ERK, (**C**) N-cadherin, β–catenin and (**D**) PD-L1 were analyzed by western blot analysis. GAPDH or Actin were used as loading control

To elucidate the role played by BM-MSCs in HNSCC TME, first we explored whether they could promote cancer cell invasion and investigated at the same time the underlying mechanisms. To this aim, we performed an invasion assay using a Boyden chamber with membrane coated with matrigel and with FaDu and SCC-011 cells plated in the upper chamber and BM-MSCs or medium with 10% FBS (control) put in the lower chamber as chemoattractant. The presence of BM-MSCs enhanced the invasiveness of tumor cells compared to control (23% for FaDu and 50% for SCC-011, *p* < 0.01) (Fig. 2A). The signaling pathways playing a crucial role in tumor progression and malignant transformation, such as the activation of AKT and ERK, were analyzed in FaDu and SCC-011 cells co-cultured with BM-MSCs. The expression levels of phosphorylated AKT and ERK were enhanced in HNSCC cells grown in presence of BM-MSCs (Fig. 2B). In addition, cancer cell exposure to stromal cells caused an increase in EMT program markers, such as N-cadherin and β-catenin in both FaDu and SCC-011 cell lines (Fig. 2C). Taken together these results indicate that BM-MSCs induce a more aggressive and pro-metastatic phenotype of HNSCC cells.

Several studies have indicated a strong correlation between EMT status and immune checkpoint expression in multiple solid cancers [26] including HNSCC [27]. Therefore, we analyzed whether the communication between BM-MSCs and HNSCC cells affected PD-L1 expression. When BM-MSCs and HNSCC cells were co-cultured each other in trans-wells higher levels of PD-L1 expression were observed in both tumor cells and BM-MSCs (Figs. 1C and 2D). Furthermore, the relevance of these results was corroborated by the evaluation of the expression of PD-L1 in two public datasets: one including 22 normal tissues and another 103 HNSCC samples. The analysis showed that the tumors expressed higher levels of mRNA PD-L1 respect to normal tissue (Fig. S1).

### Preparation and characterization of recombinant Fab’ anti-PD-L1

**Fig. 3:**
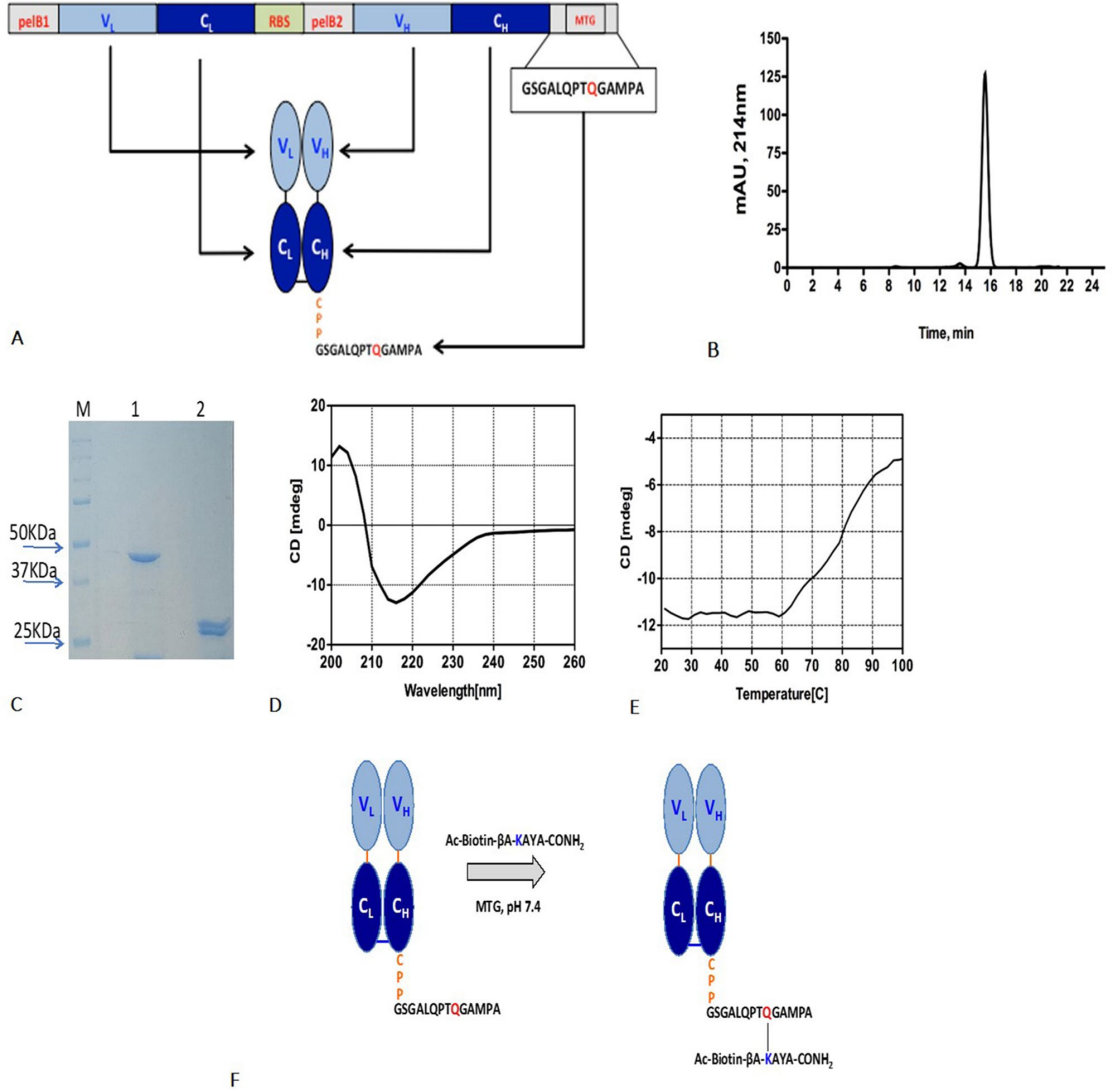
(**A**) Upper panel: schematic of the plasmid used to express the recombinant anti-PDL1 Fab’. Lower panel: schematic of the final recombinant anti-PDL1 rFab’. The VL and VH domains are in cyan while the CL and CH domains are in dark blue. CPP residues are in orange; the MTG consensus sequence is in black with the reactive glutamine in red. (**B**) Size exclusion chromatogram of anti-PDL1 rFab’ obtained with a Superdex S200/100 column. The Fab’ eluted as a single sharp peak at 15.65 mL, corresponding to a molecular weight of around 50 kDa; (**C**) 12% SDS-PAGE gel of the purified rFab’ anti-PDL1 under non-reducing (lane 1) and reducing conditions (lane 2). The molecular weight ladder (Precision Plus Protein™ All Blue Prestained Protein Standards; Biorad) is shown in lane M. Proteins were visualized by Bio-Safe Coomassie Blue stain (Biorad); (**D**) Far-UV CD spectra of anti-PDL1 rFab’ recorded at 4 µM in phosphate buffer 10 mM pH 7.5 at 20 °C; (**E**) Denaturation curves recorded at 218 nm at the temperature range from 20 °C to 100 °C. (**F**) Schematic of the reaction of enzymatic biotinylation of the anti-PDL1 rFab’ with a peptide linker sensitive to MTG that forms a covalent amide bond between the glutamine (red) side chain at the Fab C-terminus and the lysine side chain on the peptide (blue)

In order to target PD-L1 thus hampering signaling and molecular mechanisms associated with its overexpression a novel recombinant Fab’ anti-PD-L1 was developed to achieve more precise cancer targeting. Furthermore, Fabs present numerous advantages due to their smaller size, better tumor penetration and a higher tumor-to-blood ratio respect to full-length antibodies [27]. The engineered rFab’ anti-PD-L1, whose schematic representation and sequence are reported in Fig. 3A and S2 respectively, was prepared as recombinant protein in *E. Coli* and purified to homogeneity. Size exclusion chromatography confirmed homogeneity and monomericity of the purified antibody fragment (Fig. 3B). SDS-PAGE analysis under non-reducing and reducing conditions exhibited the expected bands at around 45–50 kDa (intact Fab’) and ≈ 24/26 kDa (separated light and heavy chains), respectively (Fig. 3C). Identity and integrity of rFab’ was further confirmed by LC-ESI-TOF-MS analyses under native and reducing conditions (Fig. S3), observing experimental MWs consistent with the calculated values.

**Fig. 4:**
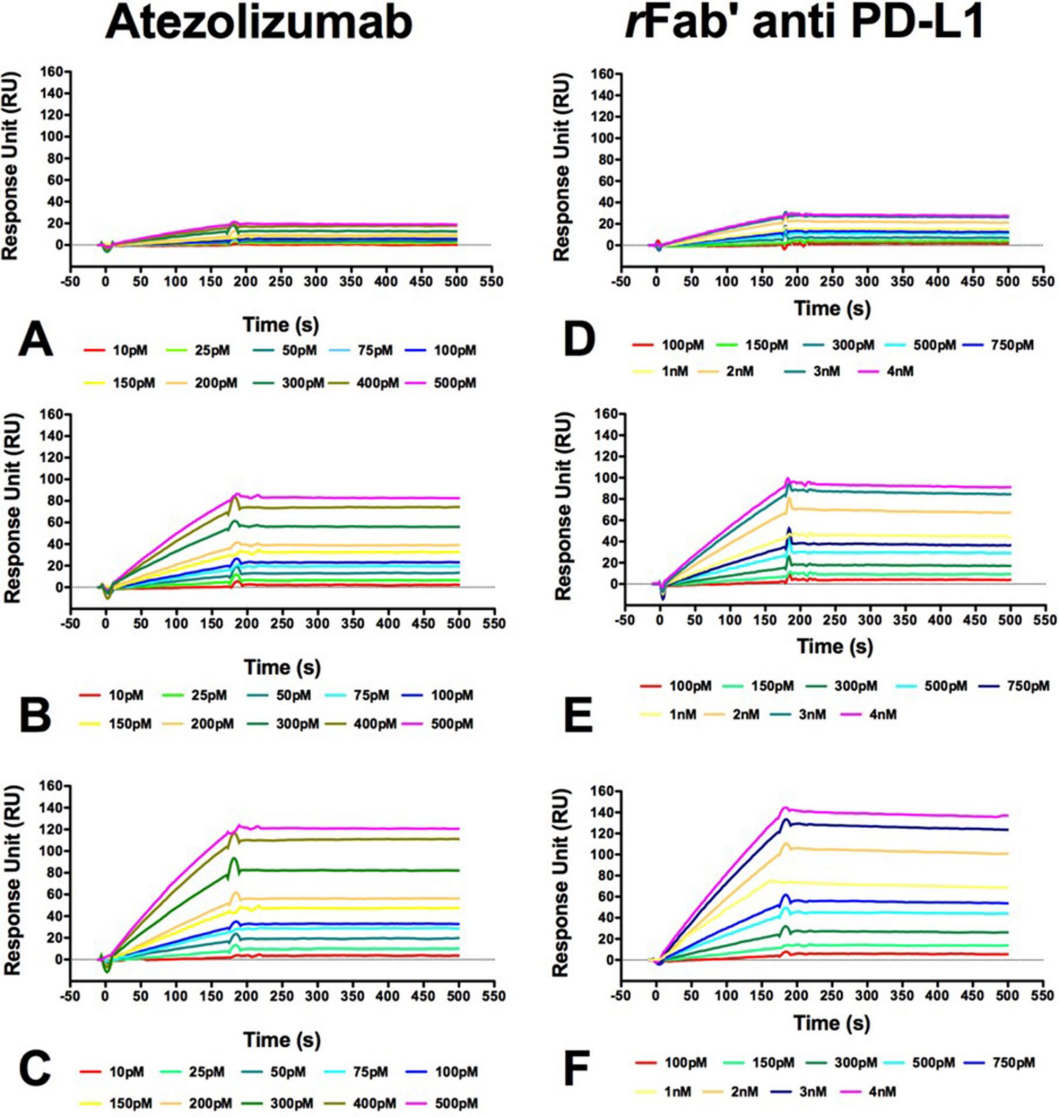
Sensorgrams overlay related to binding of Atezolizumab and rFab’ anti PD-L1 to recombinant *rh*PD-L1 protein immobilized onto a CM5 chip at a low (**A**, **D**), medium (**B**, **E**) and high (**C**, **F**) density, respectively. Binding assays were performed at 25 °C and at a constant flow rate of 20 µL/min, using HBS-EP as running buffer. By data fitting a KD of 5.8 ±2.0*10–11and 7.2+ 3.0*10–11 was calculated for rFab’ anti PD-L1 rFab’ and Atezolizumab, respectively

The proper folding and structural stability of the recombinant Fab’ were investigated by means of circular dichroism analysis. CD spectra (Fig. 3D) confirmed that the rFab’ was correctly folded, adopting secondary structures consistent with high β-sheet content. The spectrum featured a positive band at 205 nm and a negative one at 218 nm, as expected for Ig-like structures. Thermal denaturation analysis conducted monitoring the CD signal at 218 nm revealed a melting temperatures (T_m_) of 83 °C (Fig. 3E) which is similar to those reported for other similar molecules [18]. The recombinant Fab’ was tested in SPR label free assays together with the full-length ATE antibody for the binding to the antigen PD-L1 protein. Samples were injected on the CM5 sensor chip functionalized with recombinant PD-L1 at the concentrations reported in Fig. 4A. As shown, association and dissociation curves were observed at all concentrations tested and the recorded signal was concentration- and immobilization-dependent. By data fitting (Table S1) a K_D_ of 5.8 ± 2.0*10^− 11^ (averaged on medium and high immobilization experiments) was obtained for the rFab’, which, as expected, is very close to that measured with the whole antibody ATE K_D_ of 7.2 ± 3.0*10^− 11^ (averaged on the three immobilization level experiments).

### MTG-based site-specific labelling of recombinant rFab’ anti-PD-L1

To explore the potential use of the anti-PD-L1 rFab’ for the preparation of site-specifically modified Fab-drug conjugates, the antibody fragment was biotinylated at heavy chain C-terminus through a transglutamination reaction mediated by MTG. The bioconjugation reaction as monitored by LC-MS led, after 10 min, to 95% of conjugated rFab’. The resulting product, named Biotin-rFab’, purified from MTG and the free peptide, was highly pure and, as shown in Fig. S3, its experimental molecular weight was consistent with the expected mass shift (MW_exp_ 49977.51 Da/MW_theor_ 49977.36 Da).

### Detection of PD-L1 in HNSCC cells using anti-PD-L1 rFab’

**Fig. 5:**
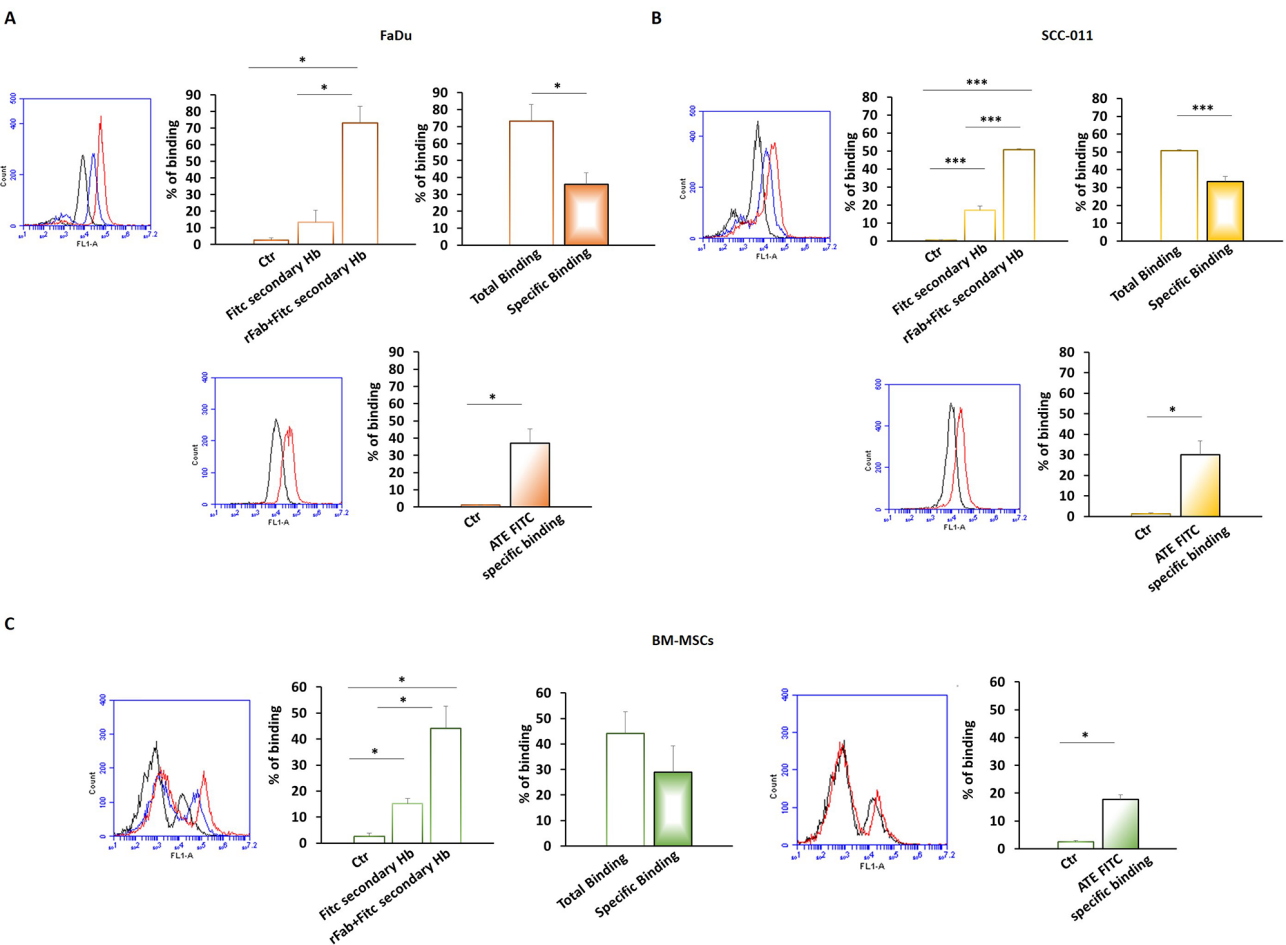
Binding assay of anti-PD-L1 rFab’ to (**A**) FaDu cells, (**B**) SCC-011 cells and (**C**) BM-MSCs by FACS analysis. The assay was performed using anti-human IgG (Fab specific) − FITC secondary antibody. Binding assay of FITC-ATE was assessed as control (*** *p* < 0.0001; ** *p* < 0.001; * *p* < 0.01)

The binding of rFab’ anti-PD-L1 to FaDu, SCC-011 and BM-MSCs was evaluated by FACS analysis in comparison with ATE. As shown in Fig. 5A and B the specific binding of rFab’ anti-PD-L1 was 36% and 33% on FaDu and SCC-011 cells, respectively, and was similar to that observed with FITC-ATE (FaDu 37%; SCC-011 30%). The binding of anti-PD-L1 rFab’ in comparison with ATE was also evaluated on BM-MSCs. In this case the specific binding of the rFab’ was 29% whereas that of FITC-ATE was only 18% (Fig. 5C).

**Fig. 6:**
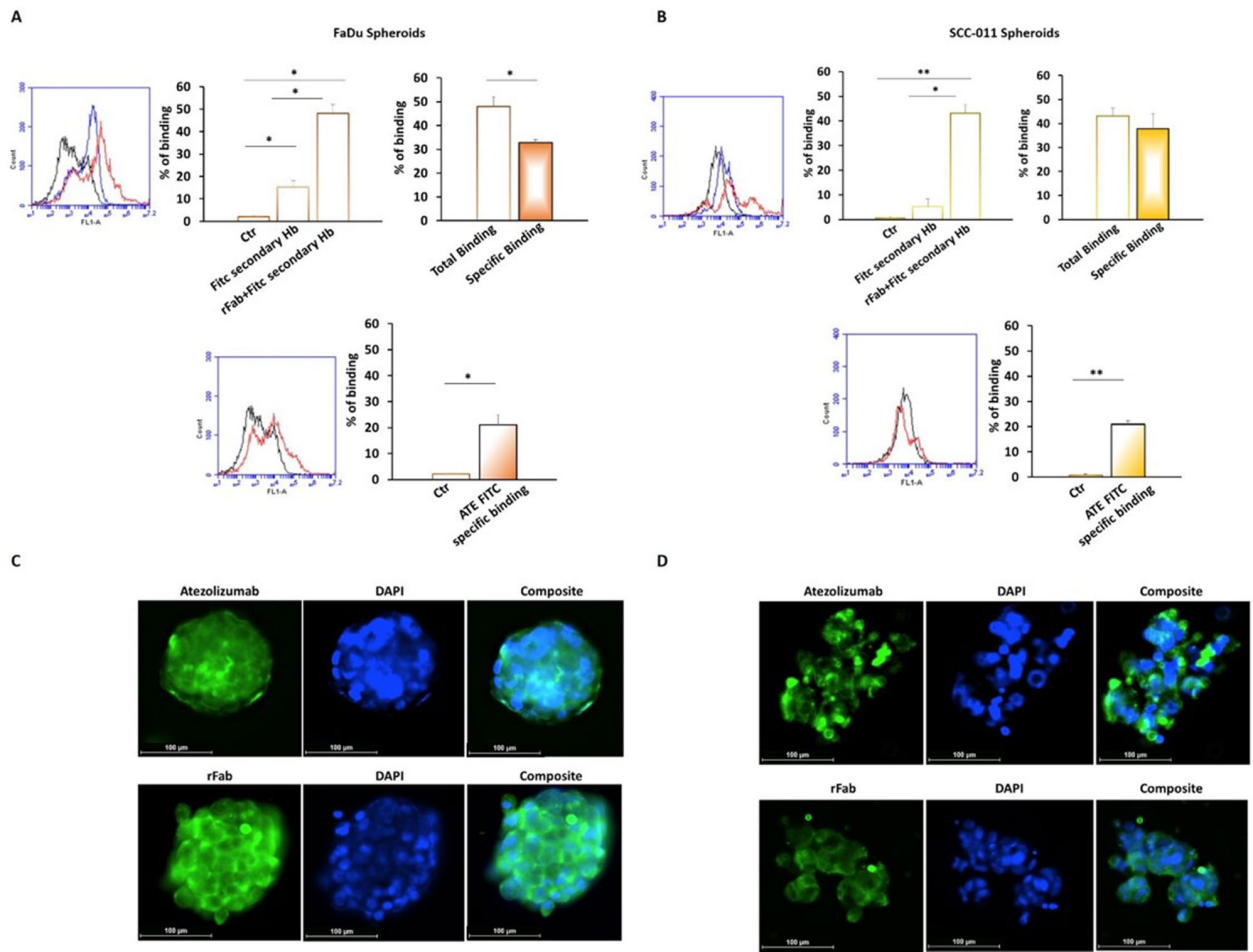
Binding assay of anti-PD-L1 rFab’ to FaDu and SCC-011 spheroids by (**A**) FACS analysis and (**B**) immunofluorescence. The assay was performed using anti-human IgG (Fab specific) − FITC secondary antibody. Binding assay of ATE was assessed as control (*** *p* < 0.0001; ** *p* < 0.001; * *p* < 0.01)

When the binding to tumor cells was examined on spheroids that better mimic the tumor structure, a specific binding of rFab’ anti-PD-L1 of 33% and 38% was measured on FaDu and SCC-011 cells, respectively, while 21% specific binding was observed with FITC-ATE (Fig. 6A). The ability of rFab’ anti-PD-L1 to bind the corresponding antigen on 3D HNSCC spheroids was also confirmed by immunofluorescence staining (Fig. 6B).

### The anti-PD-L1 rFab’ reduces viability and proliferation of HNSCC cells grown both in 2D and 3D

**Fig. 7:**
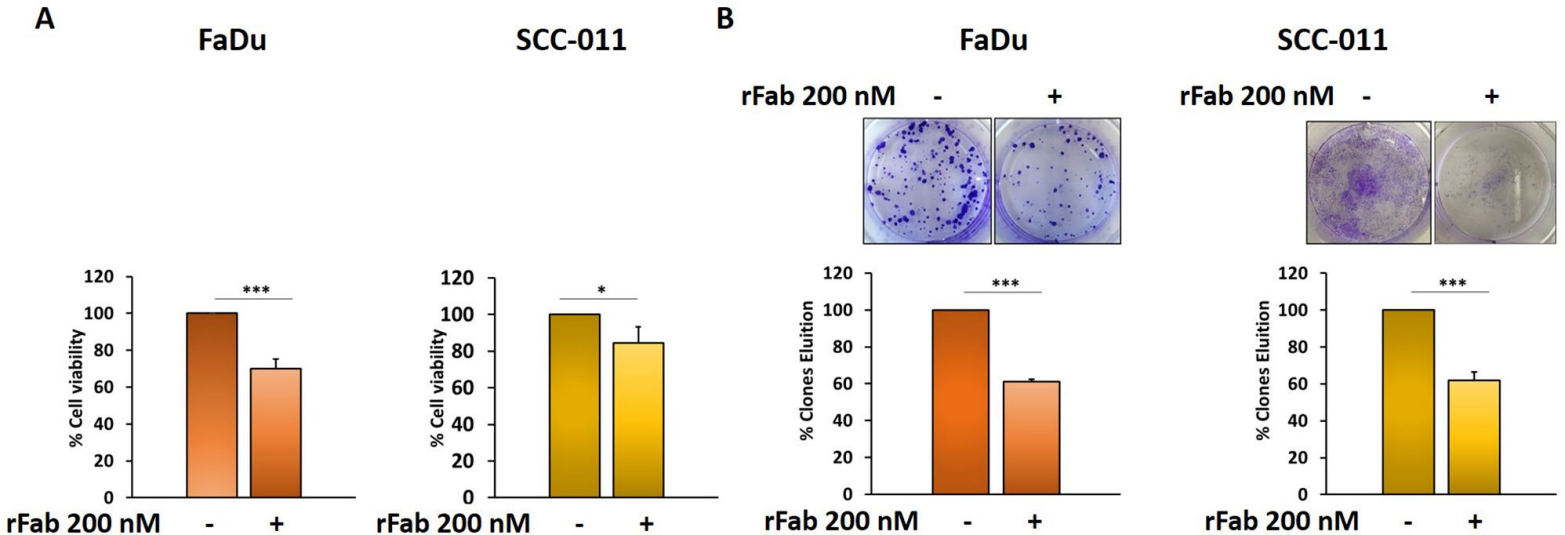
(**A**) FaDu and SCC-011 cell viability after treatment with anti-PD-L1 rFab’ (200 nM) for 72 h was evaluated by MTT assay. (**B**) FaDu and SCC-011 cell growth was tested by clonogenic assay after 10 days treatments with anti-PD-L1 rFab’ (200 nM). All the data are expressed as percentage considering the untreated cells as 100%. Bars depict mean ± SD of three independent experiments. (*** *p* < 0.0001; ** *p* < 0.001; * *p* < 0.01)

**Fig. 8:**
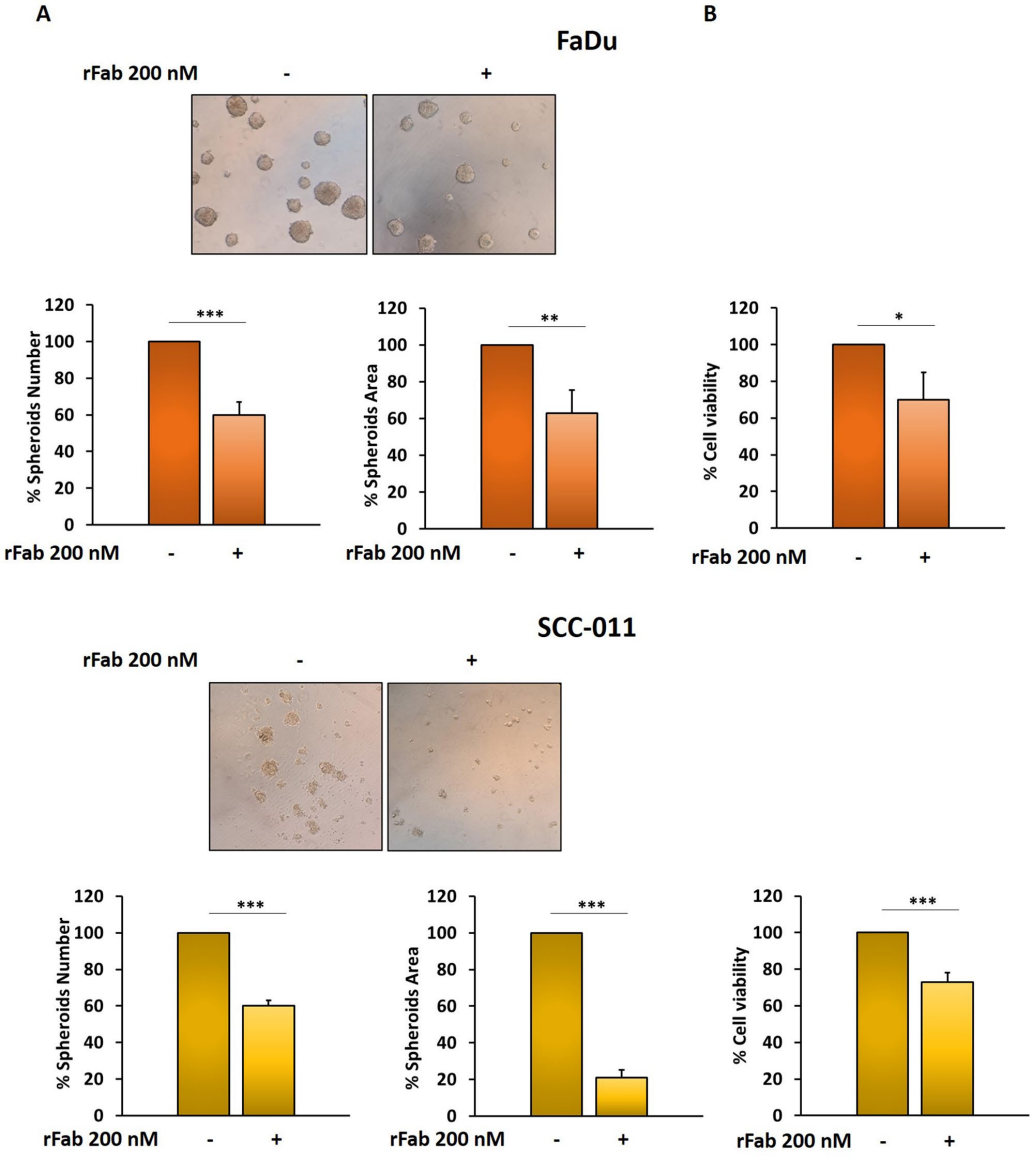
FaDu and SCC-011 cells were seeded in ultra-low attachment 96-multiwell-plates and grown in serum free DMEM supplemented with B27, bFGF (20 ng/mL) and EGF (10 ng/mL) for 48 h. Then, they were treated with anti-PD-L1 rFab’ (200 nM) for 72 h. (**A**) Spheroid formation was analyzed under a phase-contrast microscope and size and number of formed spheroids was calculated using ImageJ. (**B**) Spheroid viability was assessed by MTT 3D assay. All the data are expressed as percentage considering the untreated control cells as 100%. Bars depict mean ± SD of three independent experiments (*** *p* < 0.0001; * *p* < 0.01)

Next, the effect of anti-PD-L1 rFab’ on FaDu and SCC-011 cell viability was evaluated by MTT assay. As shown in Fig. 7A, the treatment of tumor cells with 200 nM of anti-PD-L1 rFab’ for 72 h caused a significant reduction of cell viability respect to untreated cells (FaDu 30%; SCC-011 16%). The same results were observed in BM-MSCs as reported in Fig. S4A. Moreover, the rFab’ inhibited tumor cell growth as tested by clonogenic assay after 10 days treatments (FaDu 39%; SCC-011 38%) (Fig. 7B). Interestingly, anti-PD-L1 rFab’ was able also to significantly decrease number (FaDu 40%; SCC-011 40%), size (FaDu 37%; SCC-011 79%) and viability (FaDu 30%; SCC-011 27%) of HNSCC spheroids demonstrating its efficacy on 3D models (Fig. 8A, B). In addition, MTT assays were carried out also in presence of IgG1 and ATE in tumor cells grown in 2D and 3D (Fig. S5).

### The anti-PD-L1 rFab’ hampers HNSCC cell migration, invasion and EMT program

**Fig. 9:**
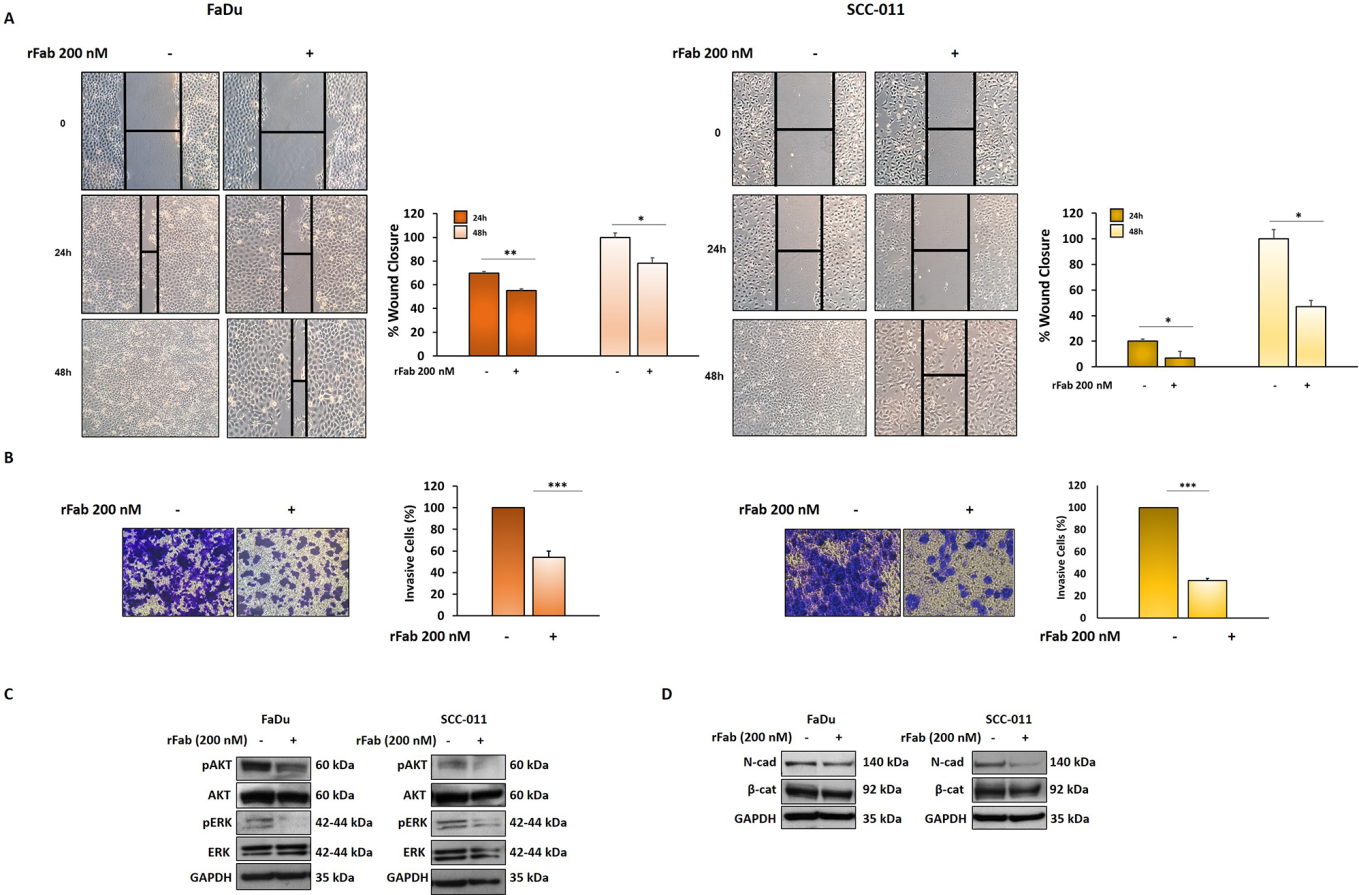
(**A**) FaDu and SCC-011 cells were scratched to create wounds and treated with anti-PD-L1 rFab’ (200 nM) for 48 h. Each scratch area was photographed at 0, 24 and 48 h. The distance between the edges of the scratch was measured by ImageJ, the average distance was quantified and the extent of wound closure was determined as follows: Wound closure (%) = 1 − (wound width tx/wound width t0) × 100 (** *p* < 0.001; *p < 0.01). (**B**) Invasion assay of FaDu and SCC-011 was performed using Boyden chamber containing inserts of polycarbonate membranes with 8 μm pores coated with Matrigel for 72 h. Tumor cells treated with anti-PD-L1 rFab’ (200 nM) were seeded in the upper chamber while medium supplemented with FBS 10% was put in the lower chamber as control chemoattractant. The results are expressed as percent of invading cells considering the untreated control as 100%. Bars depict mean ± SD of three independent experiments (** *p* < 0.001; * *p* < 0.01). FaDu and SCC-011 cells were treated with anti-PD-L1 rFab’ (200 nM) for 72 h and expression levels of following proteins: (**C**) pERK/ERK and pAKT/AKT, (**D**) N-cadherin and β-catenin were analyzed by western blotting; GAPDH was used as loading control

The overexpression of PD-L1 in cancer cells is correlated not only to immune evasion but also favors cell motility, migration and invasion. Recently, a bidirectional regulation between EMT and PD-L1 signaling has been observed in clinical patients with different cancers, which promotes locoregional growth and distant metastasis [28]. More specifically, Eichberger et al. [29] demonstrated that high levels of PD-L1 expression affected HNSCC spheroids spreading in ECM through EMT activation. In this scenario, we investigated the effect of the anti-PD-L1 rFab’ on FaDu and SCC-011 cell migration by wound healing assay. Anti-PD-L1 rFab’ at 200 nM caused a significant delay in wound closure compared to control (medium supplemented with 10% FBS) at 24 h and 48 h in both HNSCC cell lines. In particular, at 24 h FaDu wound closure was 55% in presence of rFab’ compared to 70% observed with control; SCC-011 wound closure was 7% in presence of anti-PD-L1 rFab’ compared to 20% observed with control; at 48 h, FaDu wound closure in presence of anti-PD-L1 rFab’ was 78% compared to 100% control; SCC-011 wound closure was 47% in presence of anti-PD-L1 rFab’ compared to 100% with control (Fig. 9A). Next, we evaluated whether anti-PD-L1 rFab’ could affect the ability of tumor cells to invade matrigel using Boyden chamber. We observed that treating tumor cells with anti-PD-L1 rFab’ for 72 h the invasiveness of both FaDu and SCC-011 cells was reduced to 46% and 66%, respectively, compared to control (Fig. 9B). These results correlated with an inhibition of the signaling pathways underlying EMT such as AKT and ERK phosphorylation and with a decrease of the mesenchymal markers N-cadherin and β-catenin (Fig. 9C, D).

### The anti-PD-L1 rFab’ potentiates cisplatin effect on HNSCC cell lines

**Fig. 10:**
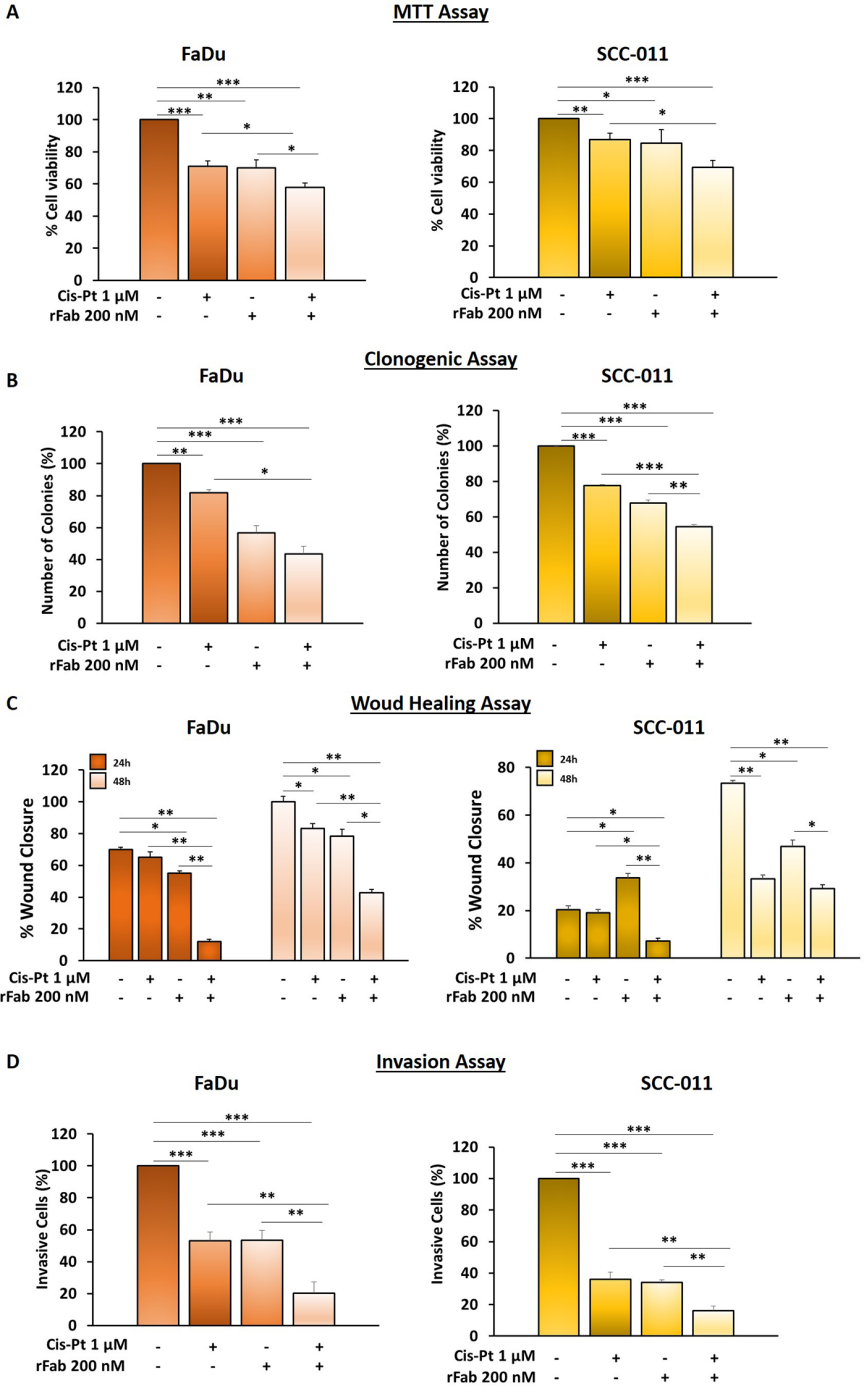
FaDu and SCC-011 cells were treated with Cis-Pt (1 µM), anti-PD-L1 rFab’ (200 nM) or the combination of the two drugs for 72 h and (**A**) cell viability was assessed by MTT assay; (**B**) cell proliferation was assessed by clonogenic assay; (**C**) cell migration was assessed by wound healing assay at 24 h and 48 h; (**D**) invasion assay of was performed using Boyden chamber containing inserts of polycarbonate membranes with 8 μm pores coated with Matrigel. All the data are expressed as percentage considering the untreated control cells as 100%. Bars depict mean ± SD of three independent experiments. (*** *p* < 0.0001; ** *p* < 0.001; * *p* < 0.01)

To evaluate whether the combined treatment of cancer cells with Cis-Pt and anti-PD-L1 rFab’ may have greater efficacy compared to single treatment, we tested both drugs on cell viability and growth using MTT and clonogenic assays, respectively. FaDu, SCC-011 and BM-MSCs were treated with Cis-Pt (1 µM) alone and in combination with anti-PD-L1 rFab’ (200 nM) for 72 h. As shown in Fig. 10A and Fig. S4B, we found that addition of anti-PD-L1 rFab’ to chemotherapy caused a significant reduction of cell viability (FaDu 42%; SCC-011 31%; BM-MSCs 33%) compared to single treatment with Cis-Pt (FaDu 29%; SCC-011 13%; BM-MSCs 12%), the viability of untreated cells was considered as 100%. Furthermore, the ability of anti-PD-L1 rFab’ to sensitize FaDu and SCC-011 cells to Cis-Pt (1 µM) was evaluated by a clonogenic assay, which showed that clone proliferation was drastically reduced when tumor cells were treated with both drugs (Fig. 10B).

**Fig. 11:**
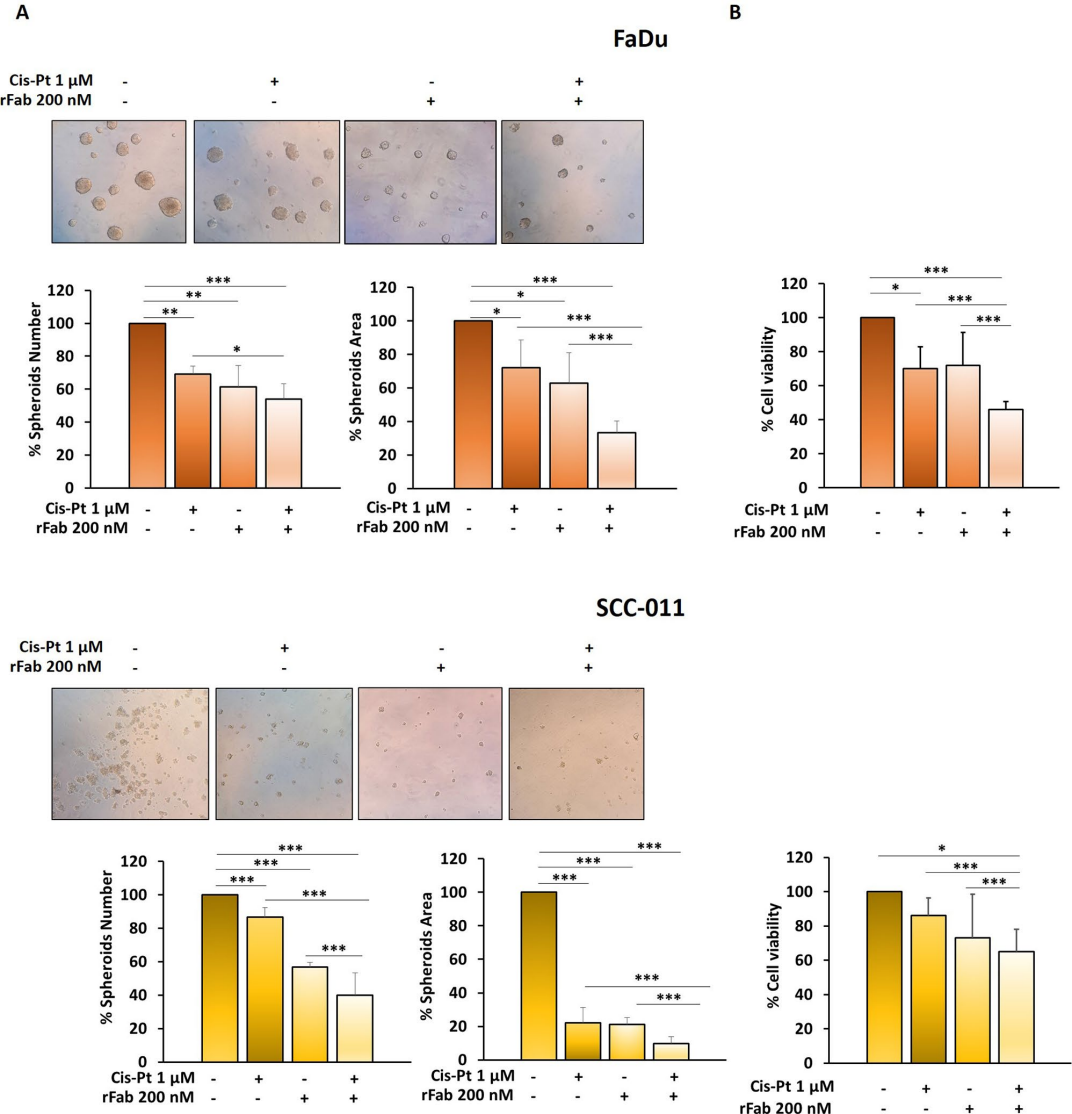
Spheroids from FaDu and SCC-011 were treated with Cis-Pt (1 µM), anti-PD-L1 rFab’ (200 nM) or the combination of the two drugs for 72 h and (**A**) spheroid formation was analyzed under a phase-contrast microscope and size and number of formed spheroids was calculated using ImageJ; (**B**) spheroid viability was assessed by MTT 3D assay. All the data are expressed as percentage considering the untreated control cells as 100%. Bars depict mean ± SD of three independent experiments (*** *p* < 0.0001; * *p* < 0.01)

Next, we investigated whether anti-PD-L1 rFab’ could potentiate the effect of Cis-Pt (1 µM) on tumor cell migration and invasion. Monolayers of FaDu and SCC-011 cells were scratched and treated with Cis-Pt (1 µM) alone and in combination with anti-PD-L1 rFab’ (200 nM) and wound closure images were taken at 0, 24, and 48 h after scratch. When HNSCC cell lines were grown in the presence of both rFab’ and Cis-Pt the closure of wound was significantly delayed compared to Cis-Pt alone (24 h: FaDu wound closure anti-PD-L1 rFab’+ Cis-Pt 12% compared to Cis-Pt alone 65%; SCC-011 wound closure anti-PD-L1 rFab’+ Cis-Pt 7% compared to Cis-Pt alone 19%; 48 h: FaDu wound closure anti-PD-L1 rFab’+ Cis-Pt 43% compared to Cis-Pt alone 83%; SCC-011 wound closure anti-PD-L1 rFab’+ Cis-Pt 29% compared to Cis-Pt alone 33%) (Fig. 10C). Similar results were obtained when HNSCC cells were allowed to invade matrigel for 72 h. Indeed, single treatment with Cis-Pt decreased the invasiveness of HNSCC cells (FaDu: reduction of 47%; SCC-011: reduction of 64%), whereas the combination of the two drugs drastically blocked it (FaDu: reduction of 80%; SCC-011: reduction of 84%) (Fig. 10D). Interestingly, anti-PD-L1 rFab’ in combination with Cis-Pt caused also a greater decrease in number, size and viability of HNSCC spheroids respect to chemotherapeutic treatment (Fig. 11). These preliminary results suggest that conjugation of cisplatin to anti-PD-L1 rFab’ might result in a potent, so far never described FDC for simultaneous immuno-cytotoxic therapy in HNSCC.

## Discussion

The TME is a complex three-dimensional ecosystem that includes the extracellular matrix in which reside tumor cells and various stromal cells (fibroblasts, macrophages, mesenchymal stromal cells, immune cells). These heterogeneous cell populations communicate with each other through multiple signals to promote tumor progression [30]. The intricate and continuous cross-talk between malignant and non-malignant cells support the emergence of phenotypes that are more aggressive in terms of resistance to therapies, metastatic spread and immune evasion [30]. In all of these processes a crucial role is played by EMT program that contributes to the creation of resistance, CSC niches, tumor cell invasiveness as well as inactivation of immune surveillance [31]. Many studies have shown that there is a strong correlation between EMT progression and the expression of immune checkpoints, especially PD-L1, in several solid tumors including HNSCC [28, 32] Ock and coworkers [27] reported that HNSCC patients showing simultaneously increased PD-L1 expression and activation of the EMT program (PD-L1+/EMT+) had a worse prognosis than PD-L1 overexpressing but EMT-negative patients (PD-L1+/EMT-), thus suggesting a key role of this program in immunotherapy resistance. Furthermore, it has been reported that the EMT score correlates with the number of invaded lymph nodes in HNSCC and can therefore be used as a predictor of lymph node metastasis in this carcinoma [33]. Interestingly, stromal cells and tumor cells are coalescing in promoting this lethal EMT/immune evasion/metastatic spread circuit through overexpression of PD-L1 and activation of AKT/MAPK signaling pathways [5]. Emerging studies focusing on the central role played by several stromal components in the regulation of HNSCC development and progression, advocate the key role of TME in providing tumor-supportive niches [34], that sustain more aggressive HNSCC phenotypes through the modulation of immune checkpoint expression [8]. Stromal cells can be recruited by different organs into TME in response to multiple stimuli, in particular inflammatory cytokines and chemokines released by tumor cells. In fact tumor can be considered “a wound that does not heal”, a scenario where they are induced to change their activity from anti-tumorigenic to pro-tumorigenic [6, 35]. In addition to the well-known role played by M2 macrophages and CAFs in the TME, also mesenchymal stromal cells can be enrolled from bone marrow and adipose tissue and to be induced to trans-differentiate into similar CAFs by tumor cells to support cancer progression [9, 25, 36–38]. In our previous study we demonstrated in vivo how vivotrack-labelled BM-MSCs are recruited in TNBC xenografts and contribute to lung metastasis formation [25]. Noteworthy, it has been found that MSC isolated from HNSCC specimens blocked T-cell proliferation similar to naive BM-MSCs and their frequency directly correlated with tumor size and inversely correlates with the numbers of tumor-infiltrating leucocytes, suggesting their crucial role in tumor escape, growth, and progression of disease [12]. Many studies shed light on the mediators involved in the cross-talk between MSCs and HNSCC cells and how this bi-directional communication impact on HNSCC [10]. Watts et al. demonstrated that BM-MSCs residing in HNSCC migrate towards TME in response to platelet derived growth factor-AA (PDGF-AA) and IL-6 produced by tumor cells [39]. In particular, MSCs isolated and characterized from malignant tissues of patients with HNSCC constitutively produced high amounts of molecules such as IL-6, IL-8 and SDF-1α and were able to enhance growth of FaDu xenografts likewise BM-MSCs when coinjected with tumor cells in nude mice [40]. Furthermore, it has been observed that BM-MSCs promoted through IL-6 proliferation, invasion, EMT, and paclitaxel resistance of HNSCC cells in vitro and in vivo [41]. Recently, Wang et al. reported that BM-MSCs co-cultured with exosomes derived from HNSCC cells are able to differentiate into CAF by involvement of miR-21 [42].

This is the basis for this study in which we demonstrate how BM-MSCs are recruited by HNSCC cells which increase their ability to migrate and invade the ECM and to change of their phenotype by inducing PD-L1 and CAF markers such as α-SMA and FAP-α. Furthermore, our results show that the cross-talk between these stromal cells and HNSCC cells, induces the EMT program, as demonstrated by the increased expression levels of N-cadherin and β-catenin via the AKT/MAPK signaling pathways, and increases the invasiveness of the tumor cells, which thus acquire a pro-metastatic phenotype.

With aim of blocking the crucial PD-L1 pro-metastatic mechanisms activated in the complex ecosystem of HNSCC, we have generated and characterized a novel anti-PD-L1 rFab’ and investigated its efficacy in reducing the aggressiveness of HNSCC in terms of cell viability, migration and invasion, as well as the signaling pathways involved. Interestingly, Fabs, due to their reduced size, have a better tumor penetration capacity and tumor-to-blood ratio in comparison with the full-length antibodies [43]. In addition they show a higher stability and better targeting capability than other fragments and lose Fc-mediated toxicities [44]. These properties make Fabs attractive as targeting agents of chemotherapeutic drugs covalently conjugated to them to overcome some of the drawbacks associated with conventional IgG-based ADCs.

Previous studies, showing that cisplatin treatments increase PD-L1 expression and optimizes immune check-point inhibition [45], that suppression of the PD-1/PD-L1 signaling improves cisplatin chemotherapy in vitro and in vivo [46] and that high expression levels of PD-1/PD-L1 confer acquired resistance to cisplatin [47]. Here, we demonstrated that the novel anti-PD-L1 rFab’ was able to hamper migration, invasion and stemness of HNSCC cell lines by decreasing expression levels of p-AKT, p-ERK and mesenchymal markers such as N-cadherin and β-catenin. Interestingly, Tran et al. showed that low doses of cisplatin may upregulate PD-L1 on tumor cells but have only modest effects on T-cell IFNγ production and may enhance antigen-specific T-cell killing of tumor cells. Furthermore, they demonstrated that in a syngeneic mouse model of HNSCC, concurrent use of cisplatin and anti–PD-L1/PD-1 delayed tumor growth and enhanced survival without significantly reducing the number or function of tumor-infiltrating immune cells or increasing cisplatin-induced toxicities [48]. Although the use of cisplatin has a strongly positive clinical impact in many tumor types, its associated side effects and drug resistance mechanisms still make it a drug with a low therapeutic index. Therefore, to improve its clinical utility, both combinational therapies and new formulations that reduce its systemic toxicity are continuously explored [49]. Currently, there are a lot of ongoing clinical trials exploring the efficacy and safety of PD-L1 blockade combined with chemotherapy, radiotherapy and targeted therapies, indeed the combination strategy is deemed as a rational and feasible approach to obtain increased treatment effects [13, 15]. In HNSCC many investigations are focusing on improving immunotherapy by combining treatments, modulating the tumor microenvironment, and utilizing nanomaterials to enhance drug delivery and immune activation [14].

## Conclusion

Our study underscores that the development of a cisplatin derivative conjugated with an anti-PD-L1 Fab would be the ideal option for HNSCC treatment, as both combination therapy and a low-toxicity chemotherapeutic formulation would be included in a single therapeutic agent. Indeed, the presence of the Fab would ensure partial masking of the cytotoxic, its targeted accumulation in PD-L1-expressing tumor tissues, and improved tumor penetration. In this study, we show that the novel anti-PD-L1 rFab’ both alone and even more in combination with cisplatin, is able to hamper malignant transformation of HNSCC cells.

Currently, research on HNSCC is centered on enhancing immunotherapy through combination therapies, modulation of the tumor microenvironment, and the use of nanomaterials for better drug delivery and immune activation. Emerging therapeutic strategies, along with a deeper understanding of patient-specific tumor biology, hold the potential to significantly improve treatment outcomes.

In the perspective of using the recombinant anti-PD-L1 Fab’ as a precursor of a FDC, it has been designed and prepared with an MTG sensitive peptide extension at the heavy chain C-terminus so as to be readily amenable to site-specific bioconjugations with amine-containing chemical moieties, as here demonstrated with the biotin-bearing linker. Remarkably, the engineered anti-PD-L1 rFab’ here presented exhibits an affinity similar to that of the parent mAb [50, 51] ensuring proper and efficient recognition of the antigen also on cell surface. Therefore, in conclusion we demonstrate the potential of anti-PD-L1 rFab’ to be conjugated to cisplatin to generate a potent FDC for immune cytotoxic therapy. All the results presented here constitute a solid basis and a strong stimulus for future in vivo studies aimed at evaluating anti-PD-L1 rFab’/cisplatin pharmacodynamic properties in a context closer to real therapeutic treatment and to possibly confirm the superior therapeutic effect of the co-administration strategy observed on cells. Looking forward, the treatment of HNSCC is advancing rapidly, driven by progresses in molecular biology, immunotherapy, and drug delivery technologies which may help in overcoming the pharmacokinetic issues predictably associated to both Fabs [52] and small sized chemoterapeutics. The future challenges in this field are still many and full of adversities. However, unconventional treatments based, as in this study, on combined treatments which are capable of attacking various survival or treatment resistance mechanisms, may pave the way to more effective, less toxic HNSCC treatments, also more suited to individual needs.

## Electronic supplementary material

Below is the link to the electronic supplementary material.

Supplementary Material 1

Supplementary Material 2

## Data Availability

No datasets were generated or analysed during the current study.

## Abbreviations

HNSCC: Head and Neck Squamous Cell Carcinoma
ECM: Extracellular Matrix
PD-1: Programmed Cell Death Protein-1
PD-L1: Programmed Cell Death Ligand-1
EMT: Epithelial to Mesenchymal Transition
AKT: Protein Kinase B
ERK: Extracellular Signal-regulated Kinase
α-SMA: α-Smooth Muscle Actin
FAP-α: Fibroblast Activated Protein -α
MAPK: Mitogen-activated Protein Kinase
BM-MSCs: Bone Marrow derived Mesenchymal Stem Cells
ICIs: Immune Checkpoint Inhibitors
TME: Tumor Microenvironment
Fab: Fragment antigen-binding region
CSCs: Cancer Stem Cells
CAFs: Cancer Associated Fibroblast
ADC: Antibody Drug Conjugate
FDC: Fab Drug Conjugate
FBS: Fetal Bovine Serum
MTG: Microbial Transglutamine
Cis-Pt: Cisplatin
ATE: Atezolizumab
BSA: Bovine Serum Albumin
CD: Circular Dichroism
SPR: Surface Plasmon Resonance
FACS: Fluorescence-Activated Cell Sorting
RT: Room Temperature
