# Supplementary material for: A novel therapeutic approach targeting PD-L1 in HNSCC and bone marrow-derived mesenchymal stem cells hampers pro-metastatic features in vitro: perspectives for blocking tumor-stroma communication and signaling
Source: Cell Commun Signal. 2025 Feb 10;23:74. doi: 10.1186/s12964-025-02073-7 (PMC11809099; doi:10.1186/s12964-025-02073-7)
Supplement: Supplementary file 1 — Supplementary Material 1 [file 12964_2025_2073_MOESM1_ESM.docx]

**Supplementary Files-Western Blot Figures:**

**A novel therapeutic approach targeting PD-L1 in HNSCC and bone marrow-derived mesenchymal stem cells hampers pro-metastatic phenotype: perspectives for blocking tumor-stroma communication and signaling**

Ylenia Ferrara, Debora Latino, Angela Costagliola di Polidoro, Angela Oliver, Annachiara Sarnella, Maria Grazia Caprio, Laura Cerchia, Menotti Ruvo, Annamaria Sandomenico, Antonella Zannetti

**Figure 1**


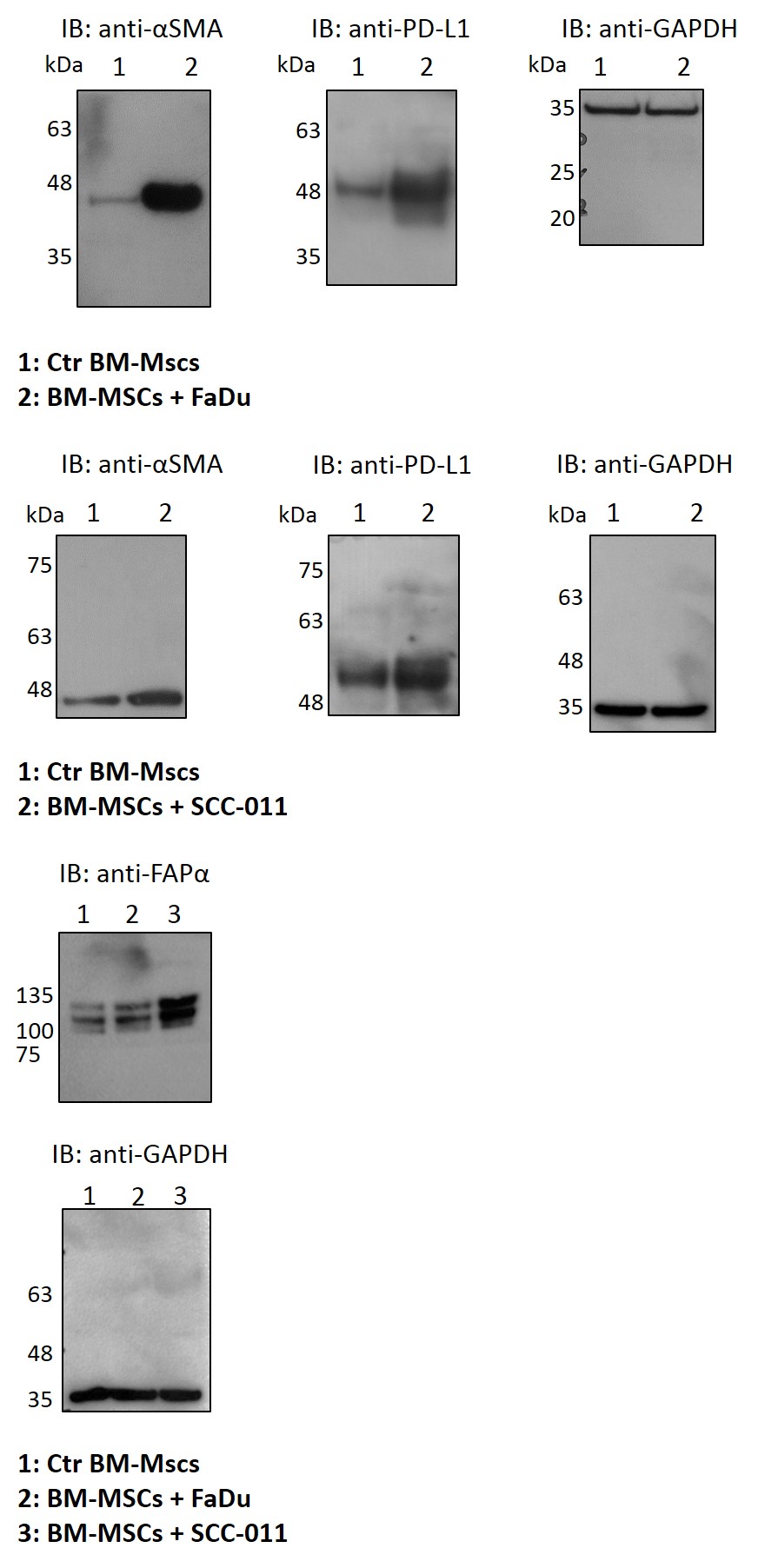


**Figure 2**


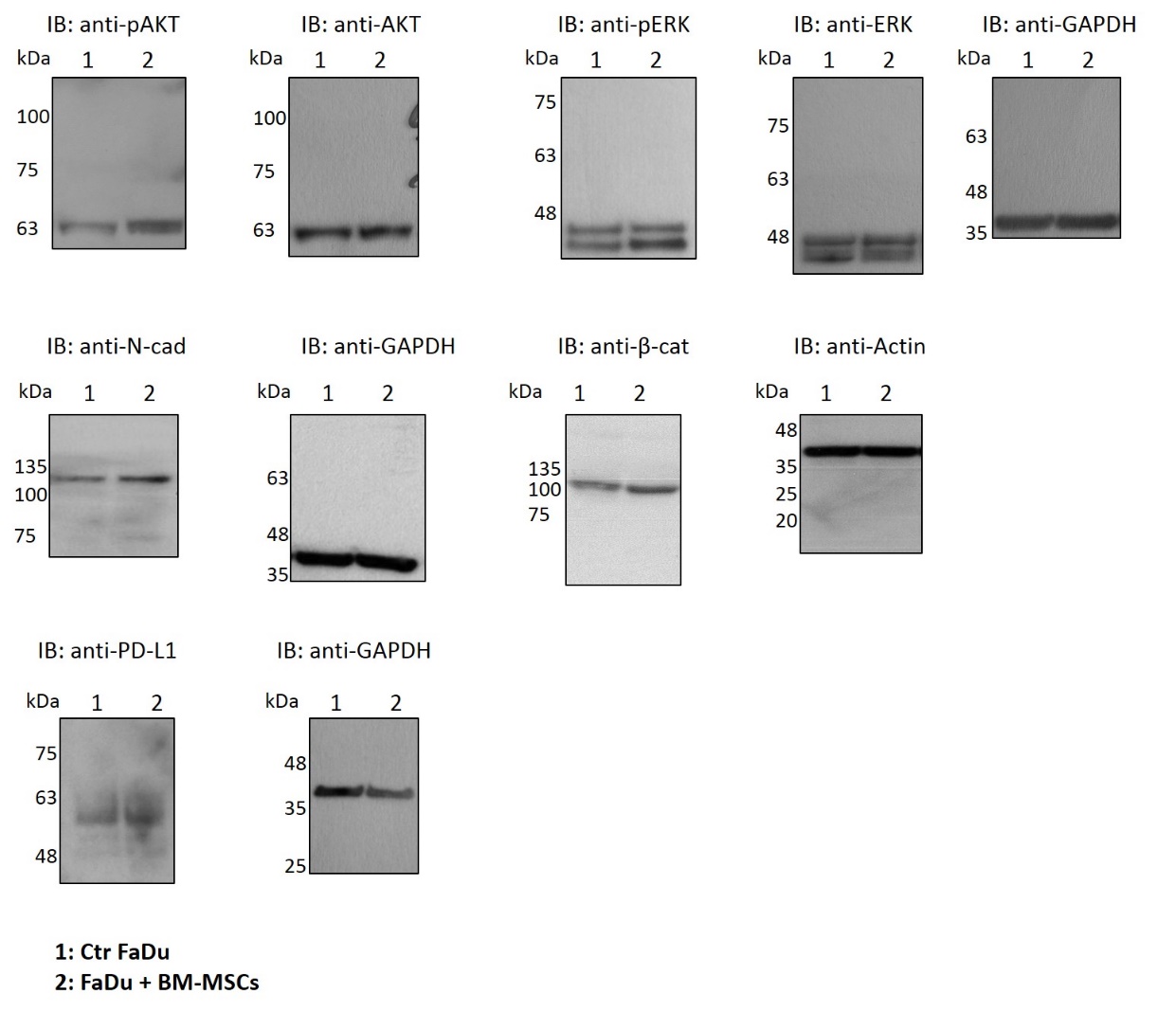


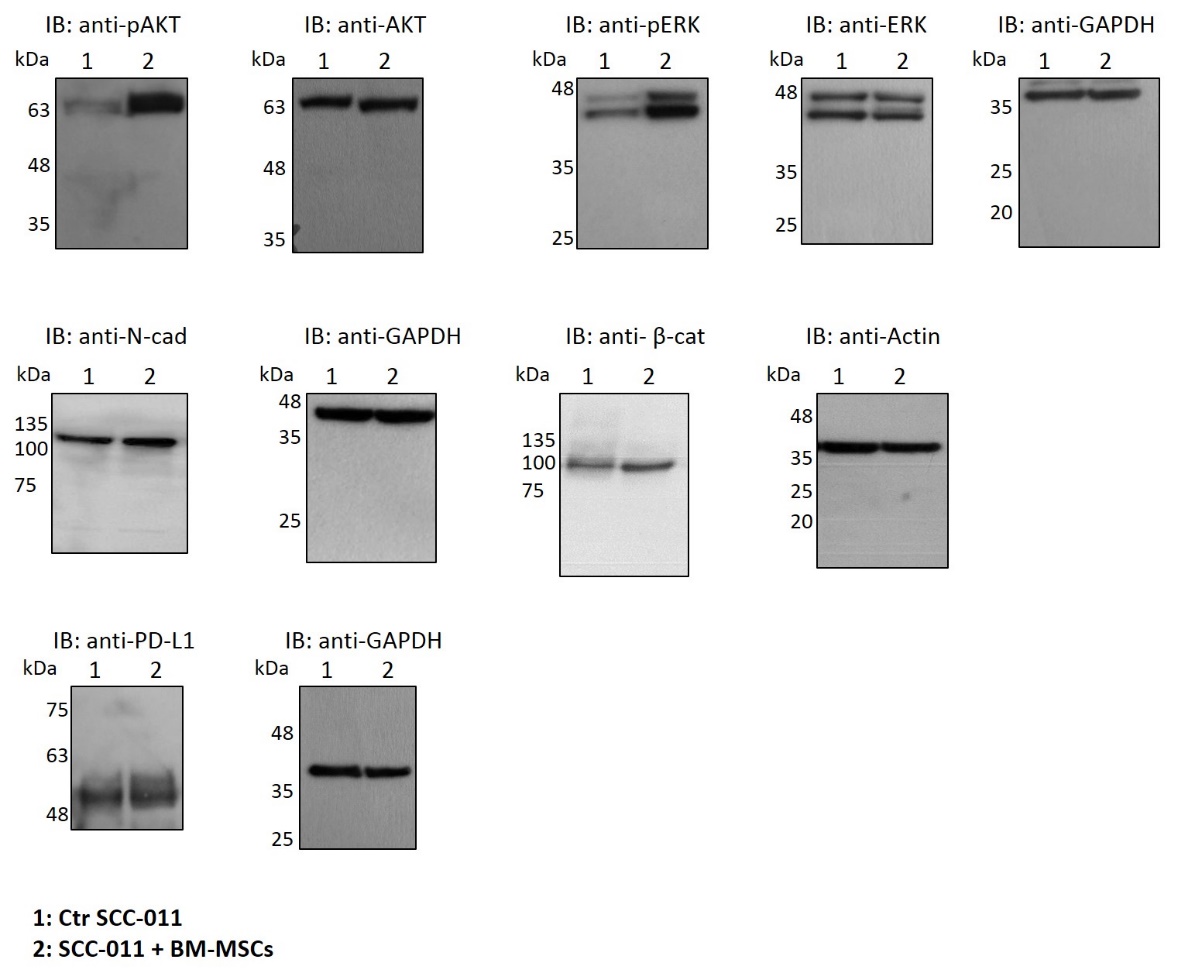


**Figure 3**

**
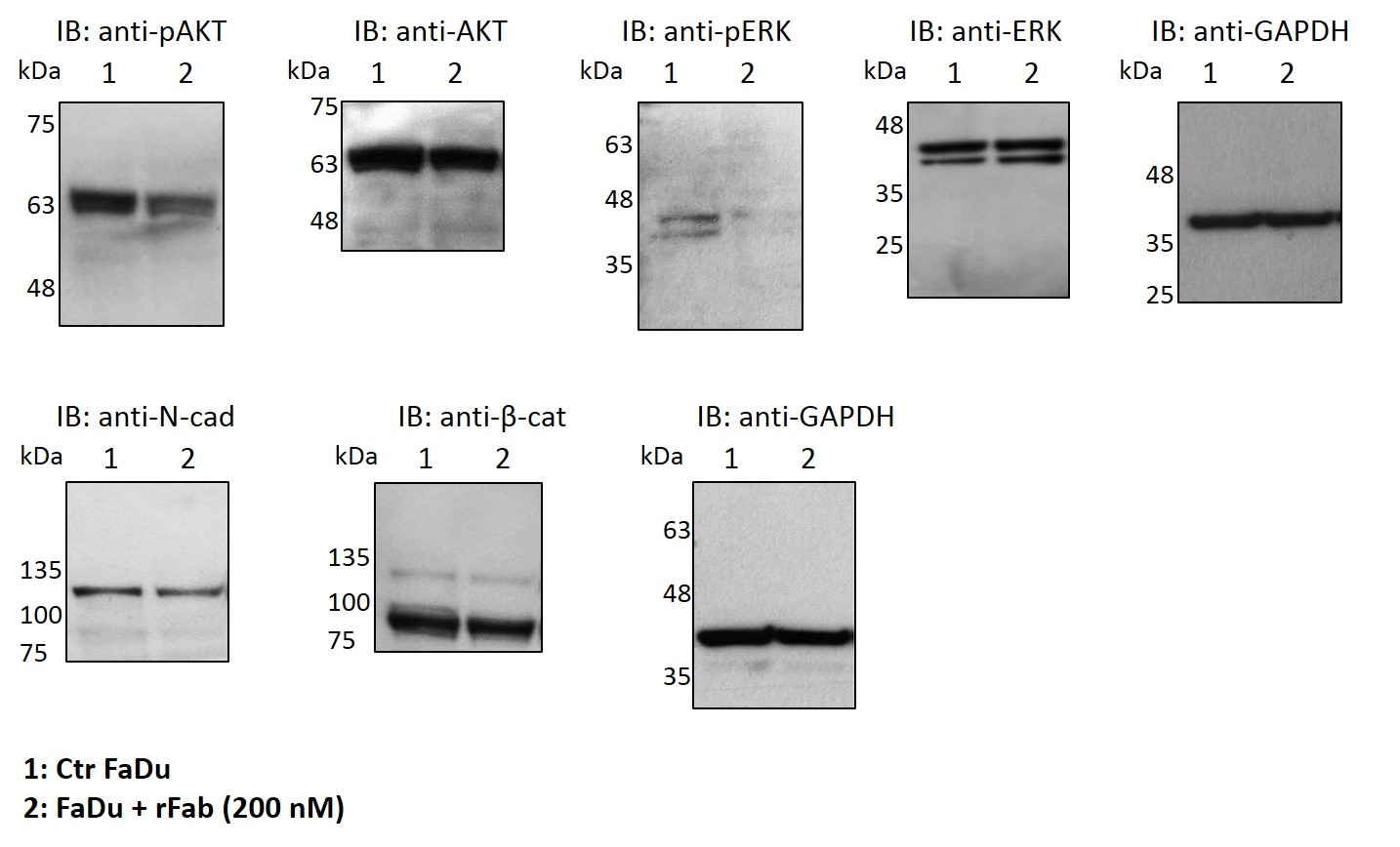
**

**
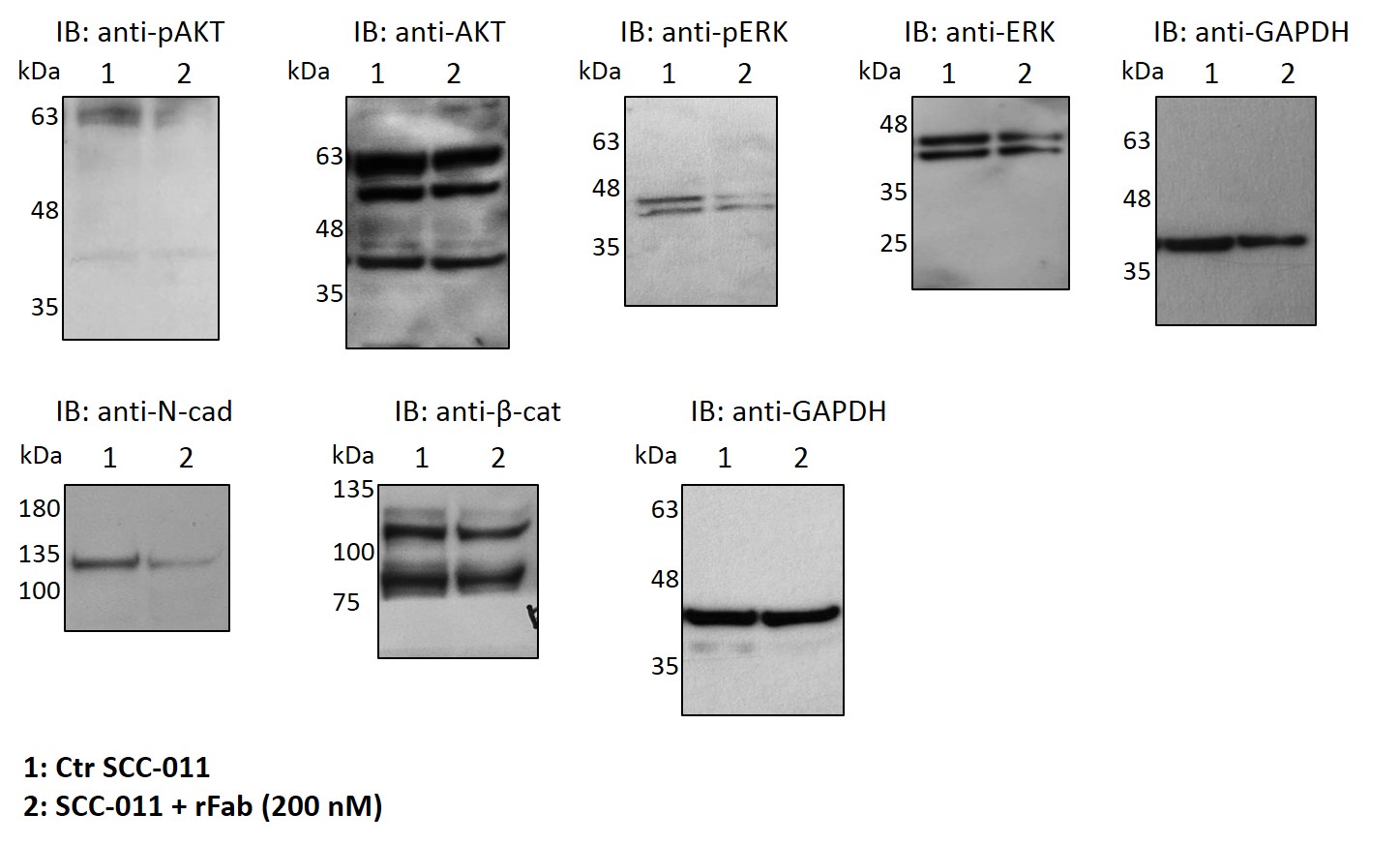
**
