# Supplementary material for: A novel therapeutic approach targeting PD-L1 in HNSCC and bone marrow-derived mesenchymal stem cells hampers pro-metastatic features in vitro: perspectives for blocking tumor-stroma communication and signaling
Source: Cell Commun Signal. 2025 Feb 10;23:74. doi: 10.1186/s12964-025-02073-7 (PMC11809099; doi:10.1186/s12964-025-02073-7)
Supplement: Supplementary file 2 — Supplementary Material 2 [file 12964_2025_2073_MOESM2_ESM.docx]

**Supplementary Material:**

**A novel therapeutic approach targeting PD-L1 in HNSCC and bone marrow-derived mesenchymal stem cells hampers pro-metastatic pro-metastatic features *in vitro*: perspectives for blocking tumor-stroma communication and signaling**

Ylenia Ferrara, Debora Latino, Angela Costagliola di Polidoro, Angela Oliver, Annachiara Sarnella, Maria Grazia Caprio, Laura Cerchia, Menotti Ruvo, Annamaria Sandomenico, Antonella Zannetti


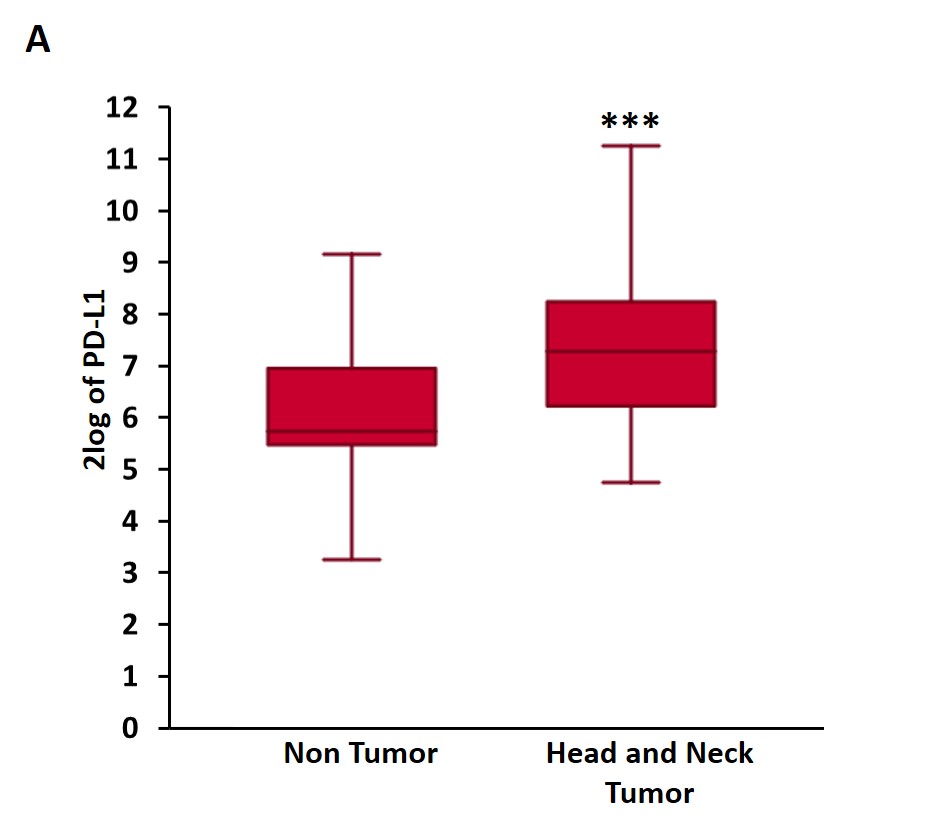


**Fig. S1** PD-L1 expression in HNSCC patients. (A) For PD-L1 mRNA expression analysis in HNSCC patients the Genomics Analysis and Visualization platform (R2: Genomics analysis and visualization platform; http:// r2. amc. nl) was used. The analysis was performed with the following datasets: GSE18674 which includes 22 human normal tissues and GSE42743 which includes 103 oral cavity tumors. The correlation was assessed by one-way analysis of variance (ANOVA), through the R2 platform and presented in box plots (*** p < 0.0001).

**
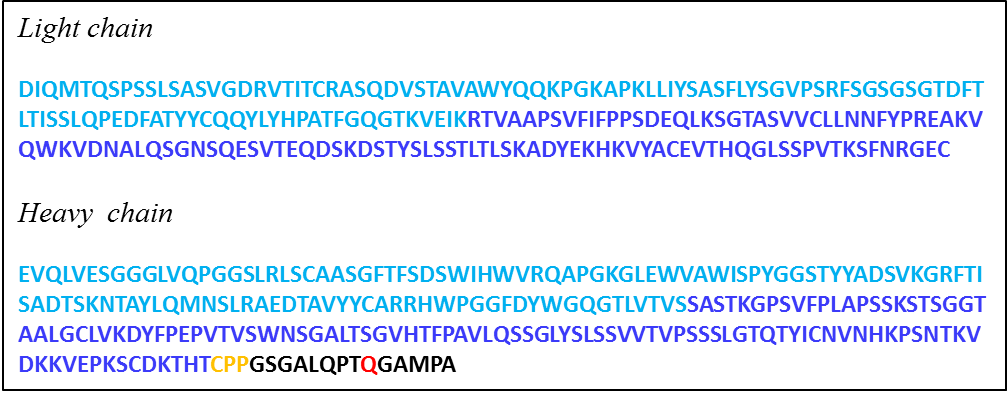
**

**Fig. S2** Sequences of light and heavy chains of recombinant anti-PDL1 Fab. Colouring of the V_L_ and V_H_ (cyan) and C_L_ and C_H_ (dark blue) domains reflect those reported in Fig. 1AB. The CPP residues are in orange, the MTG consensus sequence is in black with the reactive glutamine in red.

**
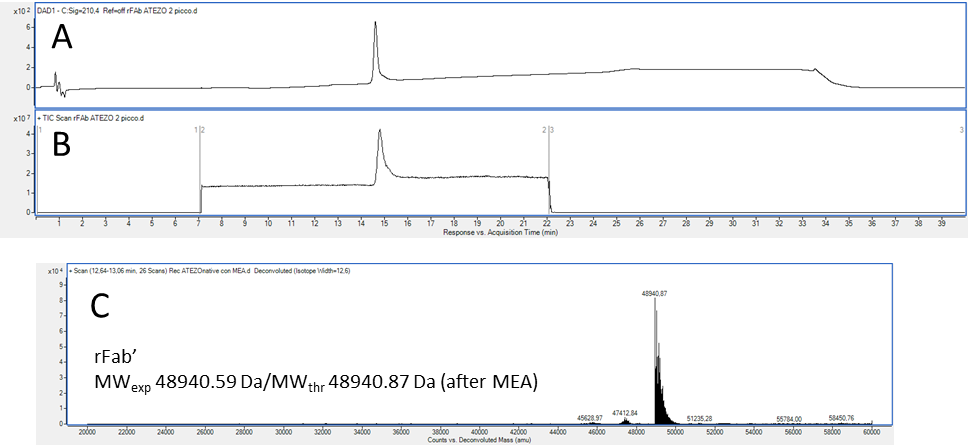
**

**Fig. S3** LC-ESI-TOF-MS analysis of the intact recombinant anti-PDL1 Fab’. (A) RP-HPLC profile; (B) TIC profile; (C) Deconvoluted mass spectrum.

| **Table S1**  **Average of kinetic parameters and apparent affinity constants of Atezolizumab and *r*Fab’ anti PD-L1 obtained by surface plasmon resonance (SPR) technique using CM5 Chips with Immobilized *rh*PD-L1 protein** | | | | |
| --- | --- | --- | --- | --- |
|  | **Azetolizumab** | | ***r*Fab’ anti PD-L1** | |
| **Level of**  **immobilization** | **KD (M)** | **Chi2** | **KD (M)** | **Chi2** |
| **LOW** | **3.26*10^-10^** | **0.0311** | **7.57*10^-10^** | **0.0548** |
| **MEDIUM** | **5.14*10^-11^** | **0.0353** | **6.56*10^-10^** | **0.0324** |
| **HIGH** | **9.34*10^-11^** | **0.0640** | **3.40*10^-10^** | **0.0328** |

**
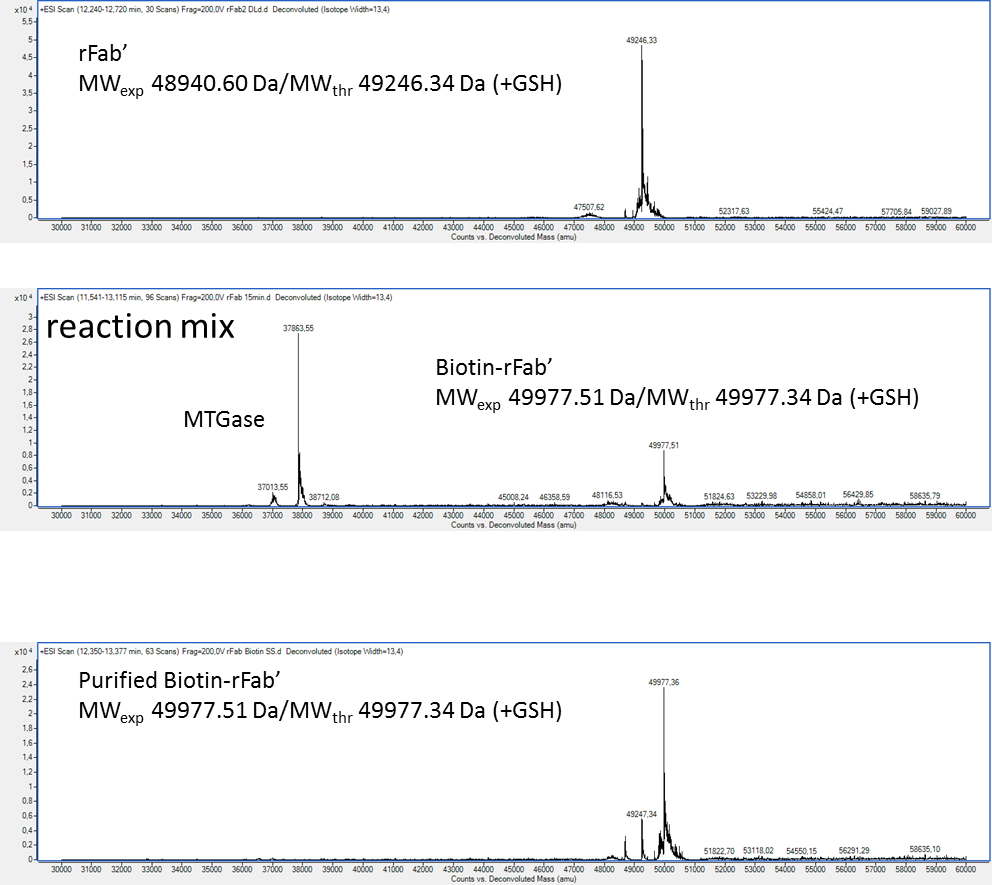
**

**Fig. S4** AS: LC-ESI-TOF-MS analysis of the bioconjugation reaction of Biotin-βΑ-KAYA-NH2 with rFab’ mediated by MTG. (a) Deconvoluted mass spectra of the intact recombinant rFab’ before conjugation and (b) in the reaction mixture after 15 minutes reaction where MTG is still present Biotin-rFab’; (c) Mass spectrum of the purified biotin-conjugated intact recombinant Fab’.

**
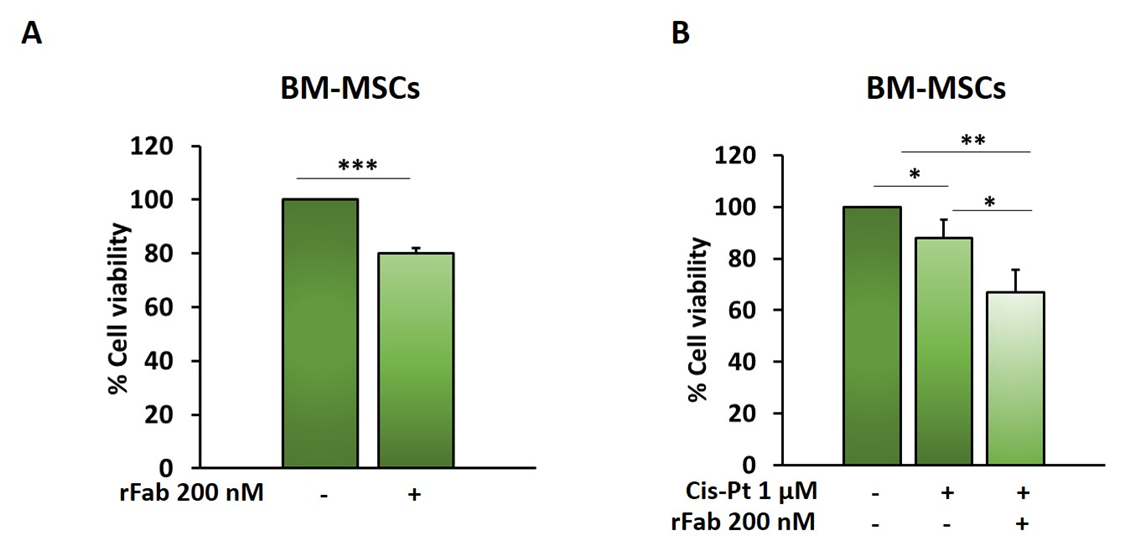
**

**Fig. S5** The anti-PD-L1 rFab’ sensitizes BM-MSCs to Cis-Pt (A) Cell viability of BM-MSCs treated with (A) anti-PD-L1 rFab’ (200 nM) for 72 hours. (B) with Cis-Pt (1 μM), anti-PD-L1 rFab’ (200 nM) or the combination of the two drugs for 72 hours by MTT assay. The data are expressed as percentage considering the untreated cells as 100%. Bars depict mean ± SD of three independent experiments. (*** p < 0.0001; ** p < 0.001; * p < 0.01).


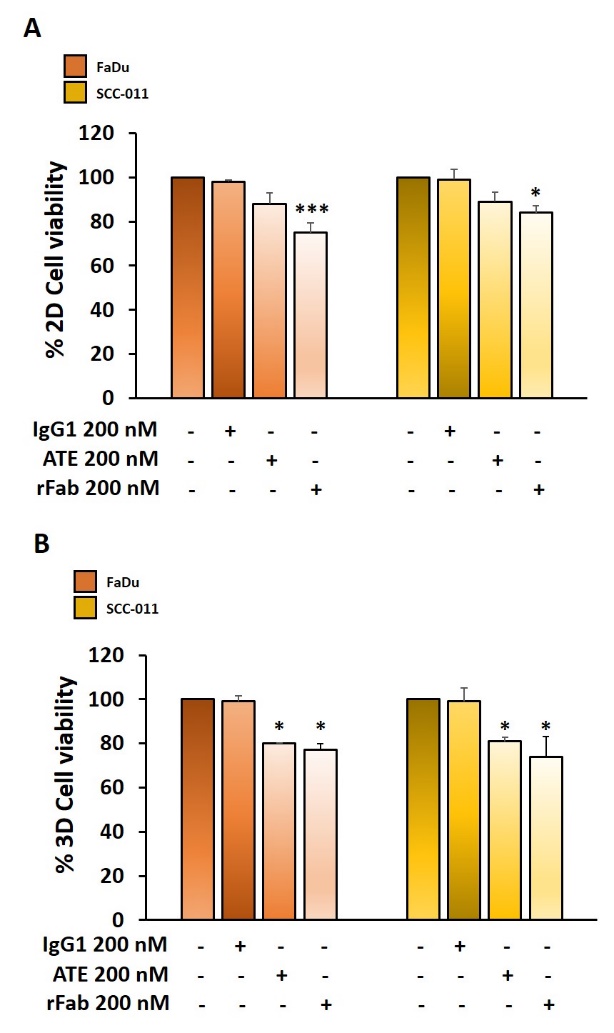


**Fig. S6** Cell viability of FaDu and SCC-011 cells (A) and spheroids (B) treated with anti-PD-L1 rFab’ (200 nM), ATE (200 nM) and IgG1 (200 nM) for 72 hours by MTT assay. The data are expressed as percentage considering the untreated cells as 100%. Bars depict mean ± SD of three independent experiments. (*** p < 0.0001; * p < 0.01).
